# Embryonic stem cells do not have a globally disrupted higher-order chromatin fiber structure

**DOI:** 10.1016/j.isci.2026.118053

**Published:** 2026-11-05

**Authors:** James Ding, Shelagh Boyle, James Allan, Nick Gilbert

**Affiliations:** 1 MRC Human Genetics Unit, Institute of Genetics and Cancer, The University of Edinburgh, Crewe Rd, Edinburgh EH4 2XU, UK

**Keywords:** Chromatin, higher-order chromatin fiber, stem cell, pluripotency, biophysical, linker histones

## Abstract

Embryonic stem cells (ESCs) are thought to maintain pluripotency through global hyper-transcription, linked to an “open” chromatin structure. To investigate this idea, we analyzed higher-order chromatin fibers from mouse ESCs, their differentiated progenitors, and terminally differentiated mouse NIH3T3 fibroblasts. Bulk chromatin composition, including protein:DNA ratio and nucleosome repeat length, varied little between the cell types; however, surprisingly, biophysical analyses, such as fluorescence recovery after photobleaching of linker histones, hydrodynamic sedimentation, and nuclease sensitivity, also showed no significant differences in the conformation of purified higher-order chromatin fibers. To better evaluate the structure of higher-order chromatin fibers observed in cells, we developed a novel technique called SPOCC (sedimentation properties of cross-linked chromatin). This approach revealed that ESCs and differentiated cells share similar bulk higher-order chromatin fiber structures, while ESCs have a slightly more disrupted structure than NIH3T3cell chromatin. These results indicate that ESC transcriptional activity and plasticity are not driven by a fundamentally “open” higher-order chromatin conformation.

## Highlights

• Comparison between higher-order chromatin fibers in mouse cells
• Bulk chromatin composition and linker histone mobility vary little between cell types
• Developed a new technique called SPOCC (sedimentation properties of cross-linked chromatin)
• Embryonic stem cells and differentiated cells have similar higher-order chromatin structure

## Graphical abstract

**Figure:**
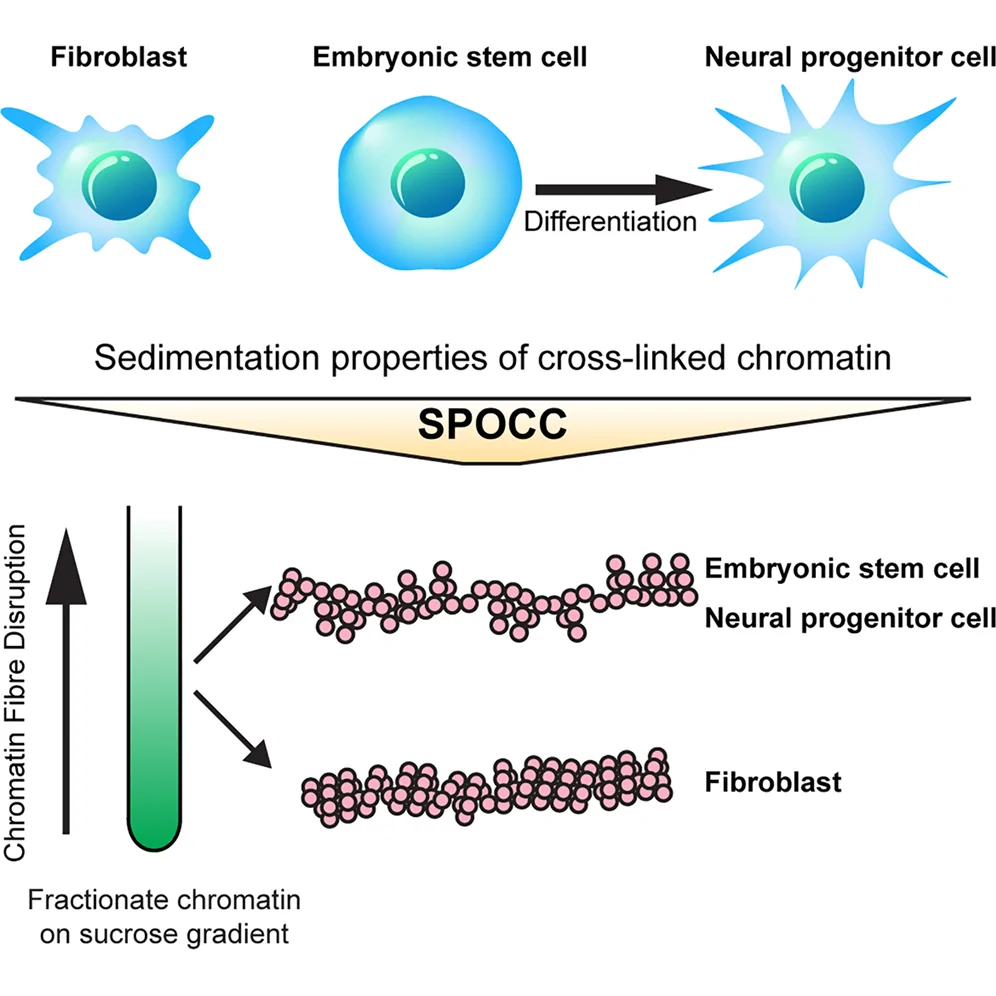

## Teaser

Epigenetics; Stem cells research; Biophysics

## Introduction

Since pluripotent mouse embryonic stem cells (ESCs) were first derived, they have been studied in detail and found to possess many distinct characteristics,^1^ including a short cell cycle,^2^ sensitivity to DNA damage,^3^ high levels of homologous recombination,^4^ and low mutation rates.^5^ In addition, stem cells express 40–60% of their genes, compared with only 10–20% of differentiated cells, and show high transcriptional heterogeneity, a property that is thought to provide them with pluripotent potential.^6^ Stem cells are also highly transcriptionally active, in a state that is described as hypertranscription,^7^ which might be important for meeting the energetic demands of rapidly growing stem and progenitor cells during development. The basis for this is not fully understood, but it has been suggested that stem cells have a unique “open” chromatin fiber structure that is both plastic and dynamic, is necessary for pluripotency,^8^ and is permissive for high levels of transcription.^9^^,^^10^^,^^11^

In eukaryotic cells, DNA is wrapped around histone proteins to form nucleosomes, which self-assemble into chromatin fibers^12^ primarily through electrostatic interactions.^13^ The nucleosome building blocks are well described,^14^ but the organization of these components into higher-order structures is less well understood,^15^ particularly how these structures might influence development and differentiation. *In vitro* studies show that nucleosomes fold to form higher-order chromatin fibers that are approximately 30 nm in diameter^16^^,^^17^ and have a helical configuration.^18^ Analogous structures are observed in regions of the genome where nucleosomes are uniformly spaced, such as at centromeric satellites,^19^ but across the majority of the genome, especially in genic regions, nucleosomes are irregularly positioned,^20^ creating disruptions in the chromatin fiber and giving it an irregular appearance.^21^ Furthermore, the often-observed images of chromatin fibers belie their dynamic and flexible structure,^22^ which is important for facilitating transcription factor binding and engagement of the transcription machinery.

Chromatin is often characterized by the nucleosome repeat length, which is dependent on the number of nucleosomes per length of DNA. Cell types with high transcriptional activity typically have more bound nucleosomes and shorter repeat lengths, as observed in *S. cerevisae*^23^ or calf cortical neurons.^24^^,^^25^ In contrast, ESCs have a repeat length of 186 bp,^26^ which is reported to increase by 5–7 bp during cell differentiation.^26^ Nucleosome positioning has also been characterized by mapping nucleosome dyads using chemical cleavage, which suggests that fragile nucleosomes can occupy previously identified nucleosome-depleted regions around transcription start sites of ESCs.^27^ These promoter proximal nucleosomes are also often composed of variant histones such as H3.3 and H2AZ^28^^,^^29^ that bind to DNA with reduced affinity^30^ and may create flexibility points in the chromatin fiber,^31^ which facilitate access to chromatin complexes^32^ and endow the chromatin fiber with an ability to be more transcriptionally dynamic, mediated by the H2AZ acidic patch.^33^

The electrostatic folding of the chromatin fiber depends on linker histones.^13^^,^^34^ Unlike core histones,^35^ linker histones are highly dynamic^36^^,^^37^ with a globular domain that positions the globule domain of the protein near the dyad^38^ and a long C-terminal tail that can shield charges along the fiber, facilitating folding. Linker histone levels affect chromatin folding: overexpression of linker histone H5 in rat sarcoma cells abrogates cell proliferation,^39^ while a reduction in linker histones promotes global^40^ and local^41^ nuclear decompaction. Furthermore, H1 mutations are found in lymphoma, and a reduction in linker histones shows an activation of stem cell genes.^42^ and C-terminal frameshift mutations in H1.4 lead to premature termination and give rise to a neurodevelopmental disorder called Rahman syndrome.^43^ The molecular mechanisms are not understood, but linker histone mutations or truncations could lead to altered protein binding, affecting chromatin fiber structure and global levels of transcription. Consistent with this notion, linker histone proteins are highly dynamic in mESCs,^9^ but with conflicting results,^44^ and this rapid mobility is often interpreted to indicate that ESCs have a unique and “open” or “loose” higher-order chromatin fiber^8^, a feature that is proposed to contribute to their developmental plasticity.^45^^,^^46^ Consistent with this notion, GFP-H2B shows greater fluctuations in stem cells than in differentiated cells, indicative of an altered nuclear architecture.^47^^,^^48^ Early microarray studies also demonstrated that ESCs are transcriptionally hyperactive, and this has been suggested to be a consequence of a “loose” chromatin organization of ESCs.^11^ Consistent with this observation, FAIRE-seq shows that stem cells have more disrupted or nucleosome-free regions than stem cells.^49^ At gross levels of nuclear organization, stem cells do appear to change architecture upon cell differentiation with a reorganization of chromocenters^9^^,^^50^ and a concomitant change in chromatin texture, which has been suggested to be influenced by the Chd1 chromatin remodeling complex.^46^ Furthermore, correlative electron spectroscopy imaging suggests that stem cells have more dispersed 10-nm-like fibers in both euchromatic^51^^,^^52^ and heterochromatic regions.^53^

To explore the “open chromatin” hypothesis, we examined bulk chromatin fibers isolated from stem cells and their differentiated progenitors using a combination of microscopic and biophysical approaches.^12^^,^^51^ Contrary to expectations, we could not find any differences between bulk higher-order chromatin fibers analyzed using biophysical sedimentation assays. Furthermore, we found that linker histones exhibit similar mobility in stem cells and differentiated cells. Our data suggest that the packaging of bulk higher-order chromatin fibers does not contribute to the hyper-transcription observed in ESCs or to their unlimited potential to differentiate.

## Results

### Sucrose gradient sedimentation discriminates between chromatin fibers of different structures

To explore how chromatin architecture might contribute to the high levels of transcription observed in mESCs and to investigate the “open chromatin” hypothesis, we studied the structure and conformation of intact chromatin fibers isolated from stem cells and differentiated cells.^8^^,^^9^^,^^46^ Sucrose gradient sedimentation, a widely used method for characterizing the biophysical properties of soluble chromatin fibers,^31^^,^^54^^,^^55^ was used to compare and contrast chromatin fiber structures. In this approach, chromatin fibers are isolated from cell nuclei by briefly digesting the chromatin with micrococcal nuclease under physiological conditions and gently releasing the soluble chromatin. After removal of nuclear debris, the chromatin is fractionated on a 6–40% sucrose gradient and centrifuged for 3 h using an SW41 rotor (Figure 1A). Under these conditions, the chromatin sediments in the sucrose gradient based on its mass and chromatin fiber structure. Larger and therefore heavier fibers sediment more rapidly, while smaller and lighter fibers sediment more slowly. Importantly, two fibers of the same mass and structure will sediment at the same rate, but if these fibers have different structures, they will sediment at different rates. Essentially, this is because the sedimentation rate of an unfolded fiber is reduced due to friction with the solvent compared with that of a more compact fiber. Previously, we showed that mouse and human satellite-containing chromatin fibers sedimented more rapidly than bulk chromatin, and using simple modeling, we argued that satellite-containing chromatin adopted a rigid, rod-like structure, while the bulk chromatin from the genome was interspersed with disruptions, making it more flexible.

**Figure 1:**
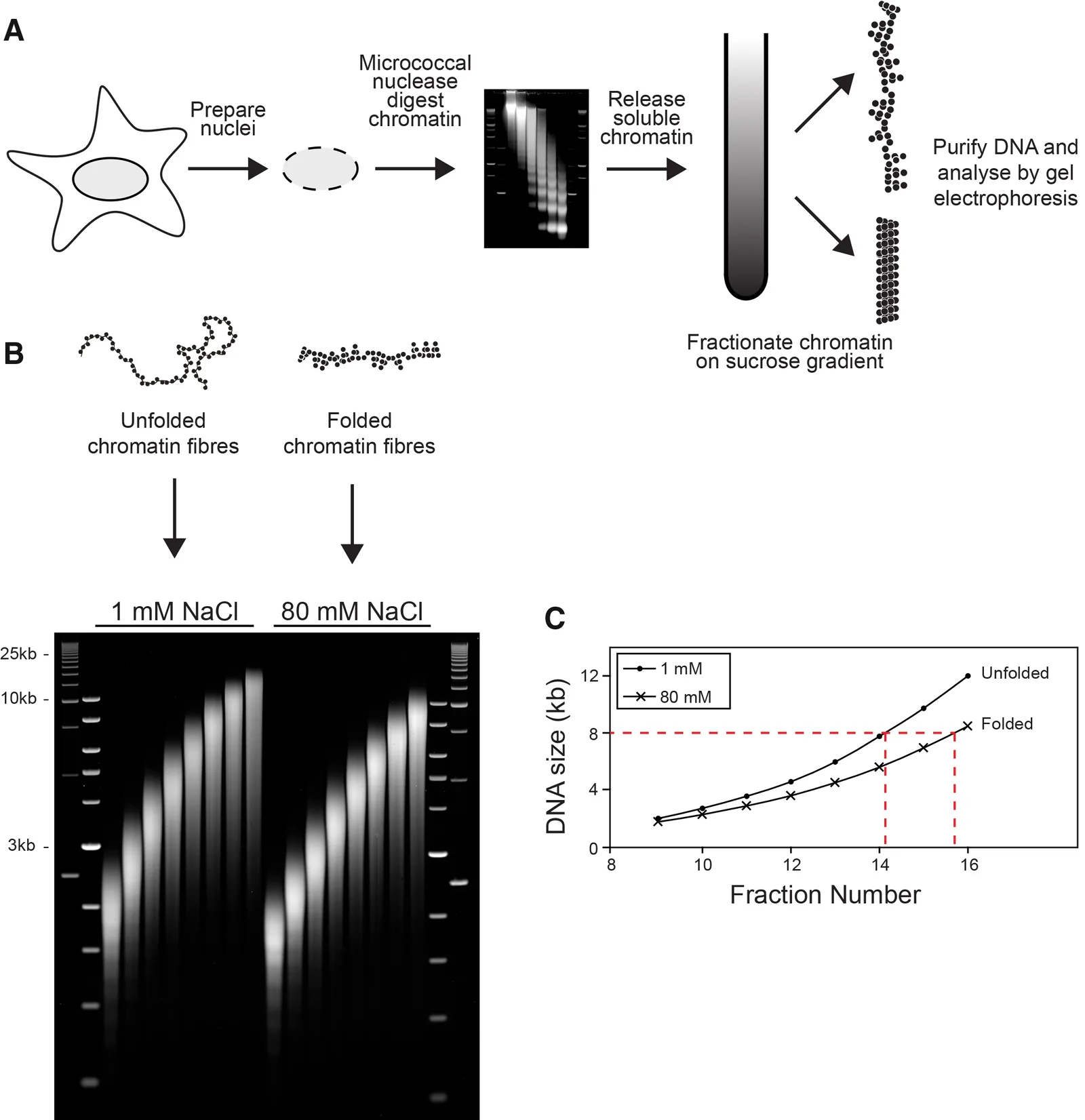
Differently folded higher-order chromatin fibers sediment at different rates (A) Schematic showing the isolation of soluble chromatin from NIH3T3 cells and fractionation in a sucrose gradient in the presence of 1 mM NaCl or 80 mM NaCl. 80 mM NaCl is equivalent to physiological-salt conditions, while chromatin fibers unfold in 1 mM salt. (B) Chromatin prepared from NIH3T3 cells and fractionated in an isokinetic sucrose gradient in the same centrifuge in the presence of either 1 mM NaCl or 80 mM NaCl. DNA was purified from individual fractions from each gradient, fractionated on an agarose gel, stained with ethidium bromide, and scanned using a Fuji FLA-5000 laser scanner. (C) Graph showing the relationship between DNA size and fraction number (sedimentation rate) for differently folded chromatin fibers. The red line shows how an 8-kb chromatin fiber with an unfolded (or disrupted) structure sediments more slowly (fraction ∼14), while an 8-kb chromatin fiber with a folded (or compact) structure sediments more rapidly (fraction ∼16). Data presented are representative of three experiments.

To demonstrate that sucrose gradients can discriminate between chromatin fibers of different structures, soluble chromatin was isolated from NIH3T3 cells and dialyzed into either a low-salt (1 mM NaCl) or physiological-salt (80 mM NaCl) buffer. Under low-salt conditions, electrostatic interactions are not effectively shielded, causing the chromatin fiber to adopt an unfolded configuration.^34^^,^^56^ After sedimentation, the chromatin was fractionated, and the DNA was purified from individual fractions and analyzed by agarose gel electrophoresis. DNA fragment size was plotted against fraction number (Figure S1C), enabling the relationship between the size and sedimentation rate to be determined. Consistent with expectations, for equivalently sized chromatin fibers (e.g., 6 kb), the sedimentation of the fiber unfolded in 1 mM NaCl was significantly retarded compared with that of fiber folded in physiological salt, demonstrating that sucrose gradient sedimentation can discriminate between chromatin fibers of different structures.

### Stem cell and differentiated cell chromatin proteins

Sucrose gradient sedimentation can discriminate between chromatin fibers of different structures (Figure 1). As the sedimentation rate is affected by the mass of the chromatin fiber, we characterized the protein composition and density of chromatin. Total nuclear proteins were extracted from mouse ESCs, differentiated ESCs, and mouse NIH3T3 embryonic fibroblasts and fractionated by SDS-PAGE (Figure 2A, left panel). Levels of core histones and linker histones were similar between the samples; therefore, to examine proteins directly bound to the chromatin fiber, soluble chromatin was isolated from cells and fractionated on a sucrose step gradient (Figure S1A).

**Figure 2:**
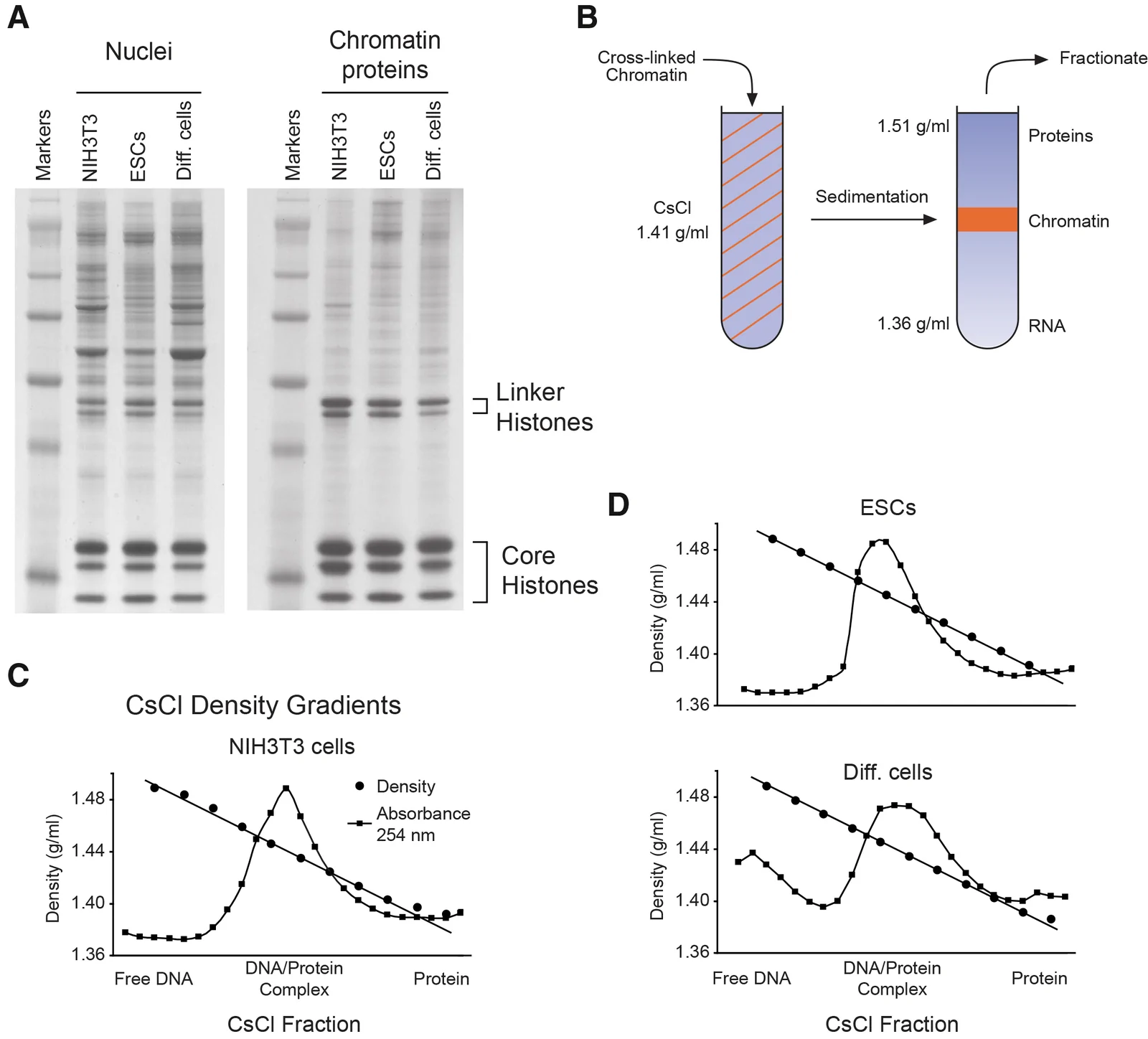
Protein and DNA composition of ESC and differentiated cell chromatin (A) Proteins were extracted from nuclei isolated from NIH3T3 cells, embryonic stem cells (ESCs), and differentiated ES cells. Chromatin proteins were extracted from chromatin that had been purified on a sucrose step gradient. Proteins were fractionated by 12% SDS-PAGE and stained with Coomassie brilliant blue. (B) Cartoon depicting the sedimentation of chromatin in a cesium chloride gradient to determine chromatin density (i.e., protein:DNA ratio). (C and D) Graphs showing the relationship between chromatin sedimentation in a cesium chloride gradient and fraction density for NIH3T3 cell, ESC, and differentiated cell chromatin. The position of chromatin (DNA/protein complex) can be estimated from the DNA absorbance of each fraction. Protein samples were prepared on two occasions. Multiple gels were run (∼8). CsCl profiles are representative of a single experiment; two experiments were carried out. These experiments are internally controlled (the density of individual fractions is measured and plotted against UV absorbance for each fraction). See also Figure S1.

To ensure that soluble chromatin fibers isolated from cells were representative of the entire genome, chromatin was prepared from ESCs, differentiated ESCs, and NIH3T3 cells. Soluble chromatin was purified on a 10–50% step gradient (Figure S1B), and the DNA was purified (Figure S1C). When the DNA probes were hybridized to mouse metaphase spreads,^55^ they gave a uniform staining pattern, indicating that the population of fibers being studied is representative of the genome (Figures S1D and S1E). After fractionation of chromatin on a step gradient (Figure S1B), proteins were isolated from the peak fraction, concentrated by ethanol precipitation, and analyzed by SDS-PAGE. As observed in the nuclei, all histone bands were very distinct (Figure 2A, right panel). However, there were slight differences in the linker histone-to-core histone ratios between independent protein preparations. As linker histones are very sensitive to degradation, these differences might be a consequence of some protein degradation. To quantitatively analyze chromatin density (essentially the protein:DNA ratio), isolated chromatin fibers were purified on sucrose step gradients (Figure S1B), dialyzed into triethanolamine buffer, and extensively cross-linked with formaldehyde (Figure 2B). After cross-linking, samples were analyzed by isopycnic centrifugation in cesium chloride gradients.^54^ By measuring the refractive index and DNA absorbance of individual fractions, the density of the purified chromatin fibers was calculated (Figures 2C and 2D). NIH3T3 cells and differentiated ESCs had very similar densities of 1.44 g/mL, while stem cell chromatin was slightly denser at 1.45 g/mL. To put this into context, this is equivalent to an increase in the nucleosome mass by 0.7% or one extra linker histone for every 12 nucleosomes.

### Characterizing bulk nucleosome repeat length

Nucleosome position influences gene transcription by modulating accessibility to the underlying DNA, while high nucleosome density correlates with high transcriptional activity.^24^^,^^25^ Multiple factors affect nucleosome repeat length, including linker histones,^57^ and their loss promotes a reduction in repeat length and global chromatin decondensation, leading to an increase in nuclear size.^40^ Furthermore, chromatin with a short repeat length does not readily bind linker histone because the altered trajectories of linker DNA sterically impair H1 binding.^58^ For this reason, the repeat lengths of stem cells and their differentiated counterparts were compared using a nuclease digestion time course. Because nucleosome repeat length is difficult to measure accurately, we have developed two new approaches to improve measurement accuracy. Historically, micrococcal nuclease was used to digest chromatin into single nucleosomes,^54^^,^^59^ but it has a tendency to nick DNA within the nucleosome and trim the nucleosome ends. We therefore used the DFF/CAD nuclease to digest chromatin^60^ (Figure 3A). The DFF/CAD nuclease precisely cuts chromatin to yield nucleosomal oligomers and trims nucleosomes to a far lesser extent. After digesting nuclei, DNA was purified and fractionated by agarose gel electrophoresis (Figures 3B and 3C). The sizes of the nucleosomal bands were accurately measured at each time point, and the data were plotted as band number versus fragment size (Figures 3D–3F). The gradient of this relationship yields the nucleosome repeat length at each digestion time point. As a second improvement, nuclei were digested with different enzyme concentrations, enabling us to extrapolate to calculate the nucleosome repeat length (Figures 3C–3E). NIH3T3 cells, stem cells, and differentiated cells all have very similar nucleosome repeat lengths of approximately 200–206 bp, consistent with what we have observed previously using micrococcal nuclease.^54^ In a previous study, Teif et al.^26^ calculated nucleosome repeat lengths from next-generation sequencing data for MEFs, ESCs, and differentiated ESCs as 191, 186, and 193 bp, respectively. This is consistent with our result, given that their experiment was performed with micrococcal nuclease, which will have trimmed the DNA fragments to some extent. Our data therefore suggest that although transcription patterns change dramatically between ESCs and differentiated ESCs, this is not accompanied by a substantive change in nucleosome repeat length and, by extension, nucleosome density.

**Figure 3:**
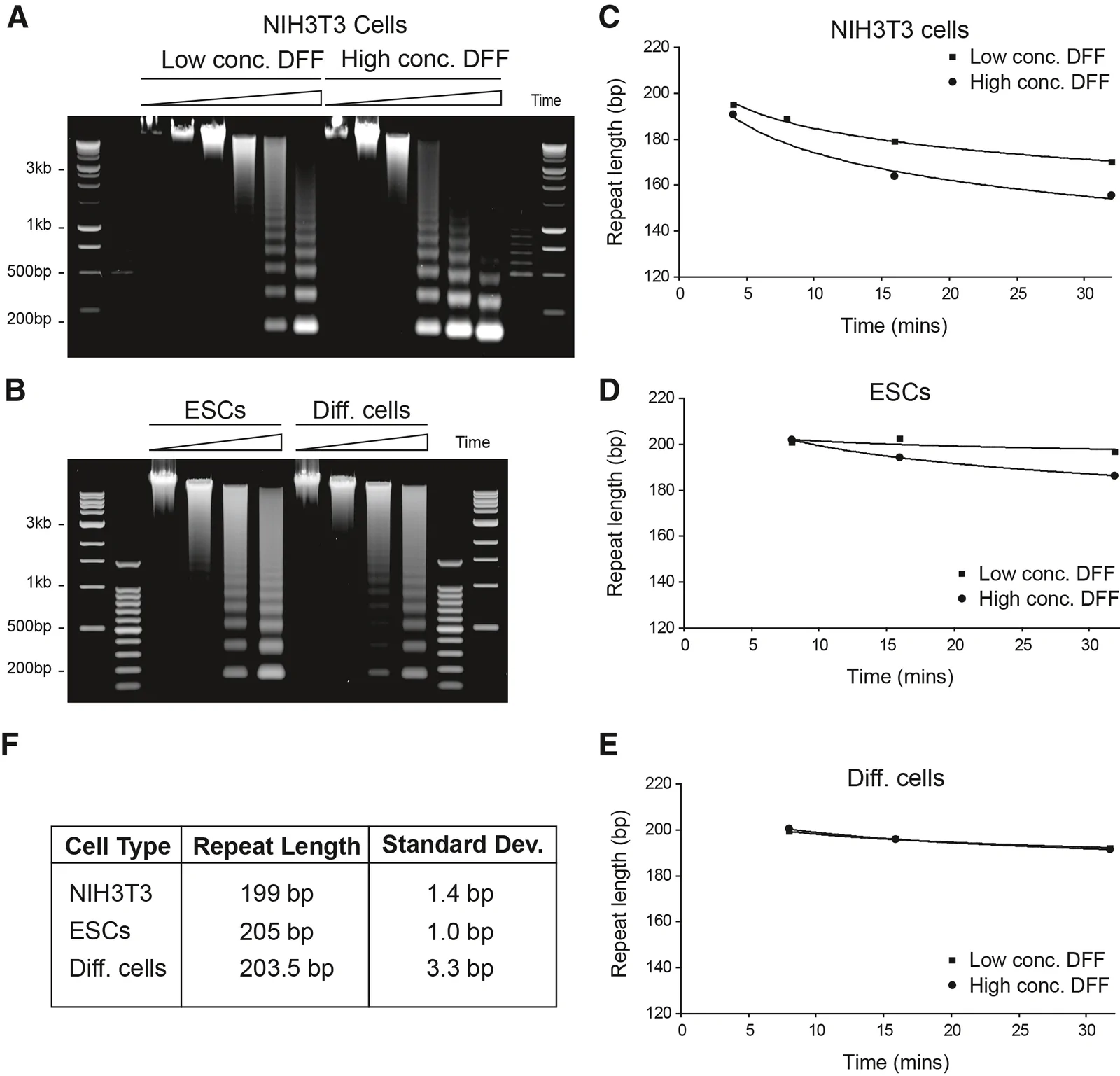
Nucleosome repeat length of ESC and differentiated cell chromatin (A and B) Nuclear samples digested with DFF nuclease, and DNA digested and fractionated on an agarose gel. (C – E) Graphs showing the nucleosome repeat length of chromatin digested for different amounts of time with different concentrations of DFF/CAD nuclease. (F) Table showing a summary of the nucleosome repeat lengths of NIH3T3 cells, ESCs, and differentiated cells. *N* = 4; median is shown with the standard deviation. Representative of four experiments. Experiments were performed with different amounts of enzyme; some experiments used DFF nuclease, while others used MNase.

### Hydrodynamic sedimentation of purified stem cell and differentiated cell chromatin

To establish whether soluble bulk higher-order chromatin fibers isolated from ESCs were more “open” or “disrupted” than chromatin from NIH3T3 fibroblasts, the hydrodynamic properties of soluble chromatin fibers isolated from these two cell types were compared. To achieve this, nuclei were briefly digested with micrococcal nuclease, and large soluble chromatin fragments were released overnight. After clarification, the soluble chromatin was centrifuged in identical 6–40% isokinetic sucrose gradients using the same rotor in TEEP_80_ buffer, which maintains chromatin in its native, physiologically folded state (Figure 1). After sedimentation, the chromatin was fractionated, DNA was purified from individual fractions, and the DNA size analyzed by agarose gel electrophoresis (Figure 4A). Plotting DNA fragment size against fraction number (Figure 4B) showed that bulk chromatin from ESC and NIH3T3 cell had equivalent sedimentation rates, suggesting that their levels of fiber compaction were very similar. Even for very large chromatin fragments of 14 kb, for which differences in structure were anticipated to be accentuated, no distinction between the two chromatin preparations was observed (Figure 4B).

**Figure 4:**
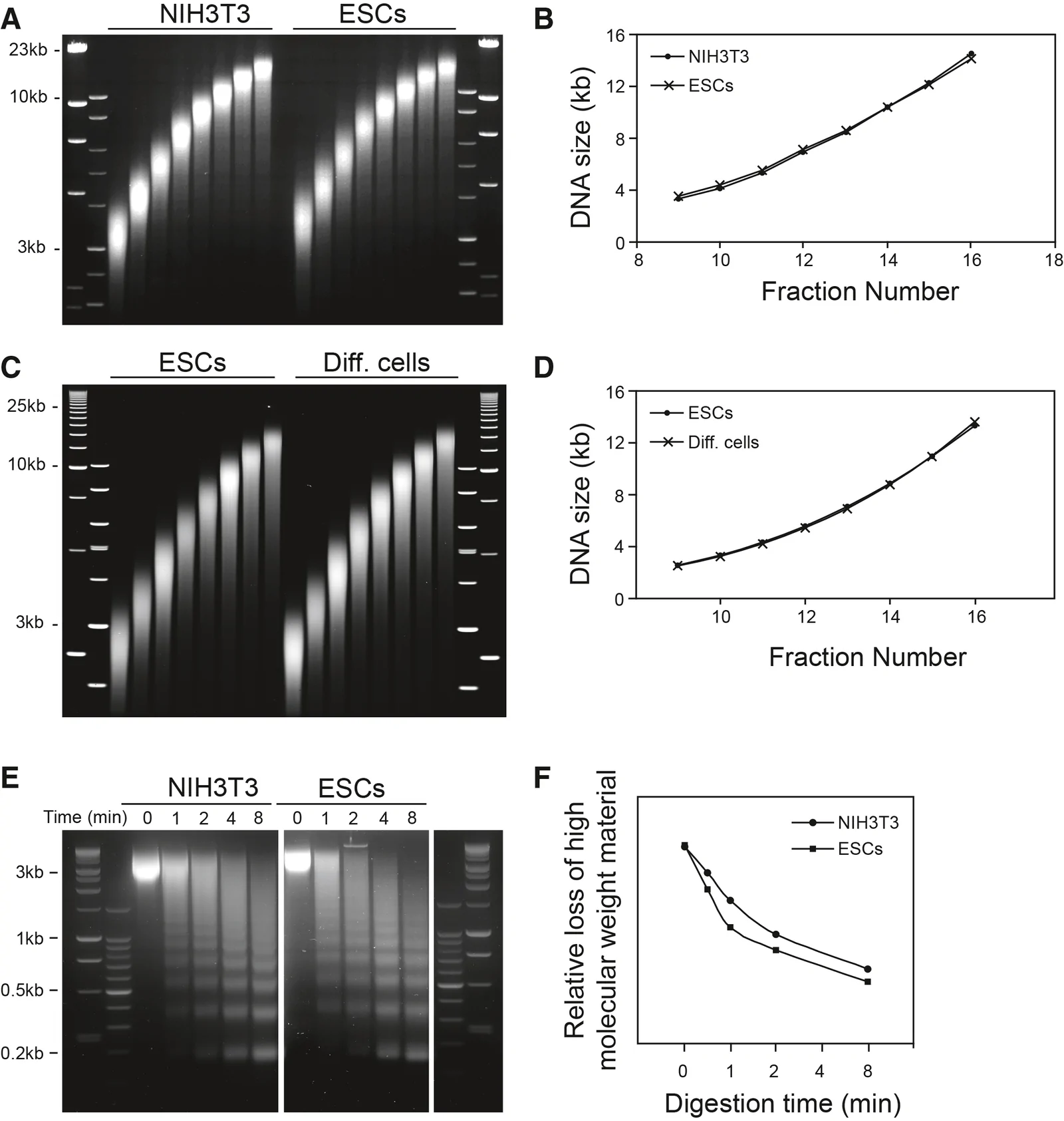
Chromatin fibers isolated from NIH3T3 cells, stem cells, and differentiated cells displaying similar hydrodynamic sedimentation properties (A) Chromatin was prepared from ES cells and NIH3T3 cells fractionated in an isokinetic sucrose gradient in the presence of 80 mM NaCl. At this salt concentration, the sucrose gradient fractionates chromatin based on its mass and higher-order chromatin fiber structure. DNA purified from individual fractions from each gradient was analyzed on an agarose gel, stained with ethidium bromide, and imaged using a laser scanner. (B) Graph showing the relationship between DNA size and fraction number (sedimentation rate) determined from the gel shown in (A) for chromatin isolated and fractionated from NIH3T3 cells and ES cells or (D) from ES cells and differentiated ES cells. (C) As for (A), but the samples were ES cells and differentiated ES cells. (D) As for (B), however, the experiment compared samples from ES cells and differentiated ES cells. (E) Soluble chromatin was isolated from NIH3T3 and ES cells and purified on a sucrose gradient. The isolated chromatin was digested with micrococcal nuclease over time, and the DNA from individual samples was purified and fractionated on an agarose gel. The gel was stained with ethidium bromide and scanned using a laser scanner. (F) Decrease in the signal intensity of the starting material analyzed and plotted against time for the two samples. Independent samples were prepared on at least two occasions, and multiple sucrose gradients were run. Data shown are representative.

Although ESCs and NIH3T3 cells have very different differentiation potentials, they are both stable, highly proliferating cell lines. It is therefore possible that, over a period of time in culture, the conformation of chromatin in differentiated NIH3T3 cells has changed to a “standard” higher-order chromatin fiber organization similar to that found in ESCs. To test this idea, the conformation of chromatin fibers from ESCs was compared directly with that of cells derived from ESCs differentiated using retinoic acid. Soluble chromatin was isolated from the cells and fractionated on a sucrose gradient, and DNA fragment sizes of individual fractions were compared (Figures 4C and 4D). Contrary to expectations, chromatin fibers isolated from ESCs and differentiated cells sedimented at similar rates, suggesting that they have very similar structures.

### Nuclease sensitivity of chromatin isolated from stem cells and differentiated cells

A detailed hydrodynamic examination of bulk higher-order chromatin fibers isolated from undifferentiated and differentiated cells failed to reveal any differences in their chromatin fiber structure (Figures 4A–4D). However, previous nuclease sensitivity studies have suggested a difference in chromatin compaction between stem cells and differentiated cells.^46^^,^^61^ Our preliminary experiments indicated that nuclease digestion of different nuclear preparations yielded unreproducible results due to variable permeability of different cell types to the enzymes. To circumvent this challenge, gradient-purified chromatin was used as the substrate for nuclease digestion.

Chromatin prepared from NIH3T3 cells and ESCs was fractionated through a sucrose gradient. Equivalently sized fractions were taken from each gradient and subjected to a micrococcal nuclease digestion time course (Figure 4E). The digestion rate was determined by quantifying the loss of high-molecular-weight DNA (>1 kb) over time (Figure 4F). Results show that ESC chromatin is digested slightly faster than NIH3T3 cell chromatin, indicating that there might be small differences in the chromatin fiber structure.

### Linker histone binding in stem cells and differentiated cells

Linker histone binding to nucleosomes shields electrostatic interactions, thereby compacting higher-order chromatin fibers *in vivo*.^13^^,^^34^^,^^62^^,^^63^ This binding predominantly occurs between the linker histone globular domain^38^^,^^64^ and the nucleosome dyad, which is abolished by mutating key residues.^65^ Previously, we used fluorescence recovery after photobleaching (FRAP) of linker histones H1 and H5 to investigate their interactions with native and unmethylated chromatin fibers.^59^ We showed that linker histones were more stably bound to chromatin in the absence of DNA methylation; however, we were unable to find a concomitant alteration in higher-order chromatin structures. Nevertheless, a reduction in nuclear linker histone levels promotes global chromatin decondensation and an increase in nuclear size,^40^ while localized H1 binding compacts chromatin and represses gene expression.^41^ As Figure 4F shows differences in nuclease sensitivity, we wondered whether this might be dependent on linker histone binding. It has also been reported that linker histone binding to stem cell chromatin is hyperdynamic, and it has been proposed that this property is a barrier to establishing higher-order chromatin structures and contributes to the maintenance of stem cell plasticity.^9^ We therefore used FRAP to investigate linker histone binding in ESCs and differentiated ESCs. Linker histones H1.4 and H5 were tagged with GFP at their N termini, adjacent to an alpha helix that interacts with linker DNA at the entry/exit point of the nucleosome,^38^ thereby minimizing any interference from GFP with the high-affinity C-terminal chromatin-binding domain of H1^66^^,^^67^ (Figure 5A). Although H1 and H5 are similar, H5 is more highly charged and binds to chromatin with higher affinity.^68^ Because of this, H5 may better discriminate between different extents of chromatin binding than H1 (Figure 5A). ESCs were transfected with GFP-H1, GFP-H5, or a binding mutant of GFP-H5 (Figure 5B), and linker histone mobility was analyzed by monitoring fluorescence recovery every 7 s after photobleaching (3 s) (Figure 5C). The recovery curves and kinetics of H1 (*t*_1/2_ = 30 s) and H5 (*t*_1/2_ = 42 s) in ESCs (Figure 3D) were similar to those we observed previously.^59^ FRAP analysis showed that H1 mobility was identical in ESCs and differentiated ESCs. In contrast, the H5-binding mutant was exceedingly mobile (*t*_1/2_ = 6 s). Histone H5 appeared to be more mobile (*t*_1/2_ = 33 s) in differentiated stem cells than in ESCs (*t*_1/2_ = 42 s); however, as the initial rates of recovery (2.7–2.9 relative fluorescence units per second) were very similar between cell types, these results indicate that linker histone mobility did not significantly vary between stem cells and differentiated cells, suggesting that differences in linker histone binding would be unlikely to be a major determinant in higher-order chromatin fiber compaction.

**Figure 5:**
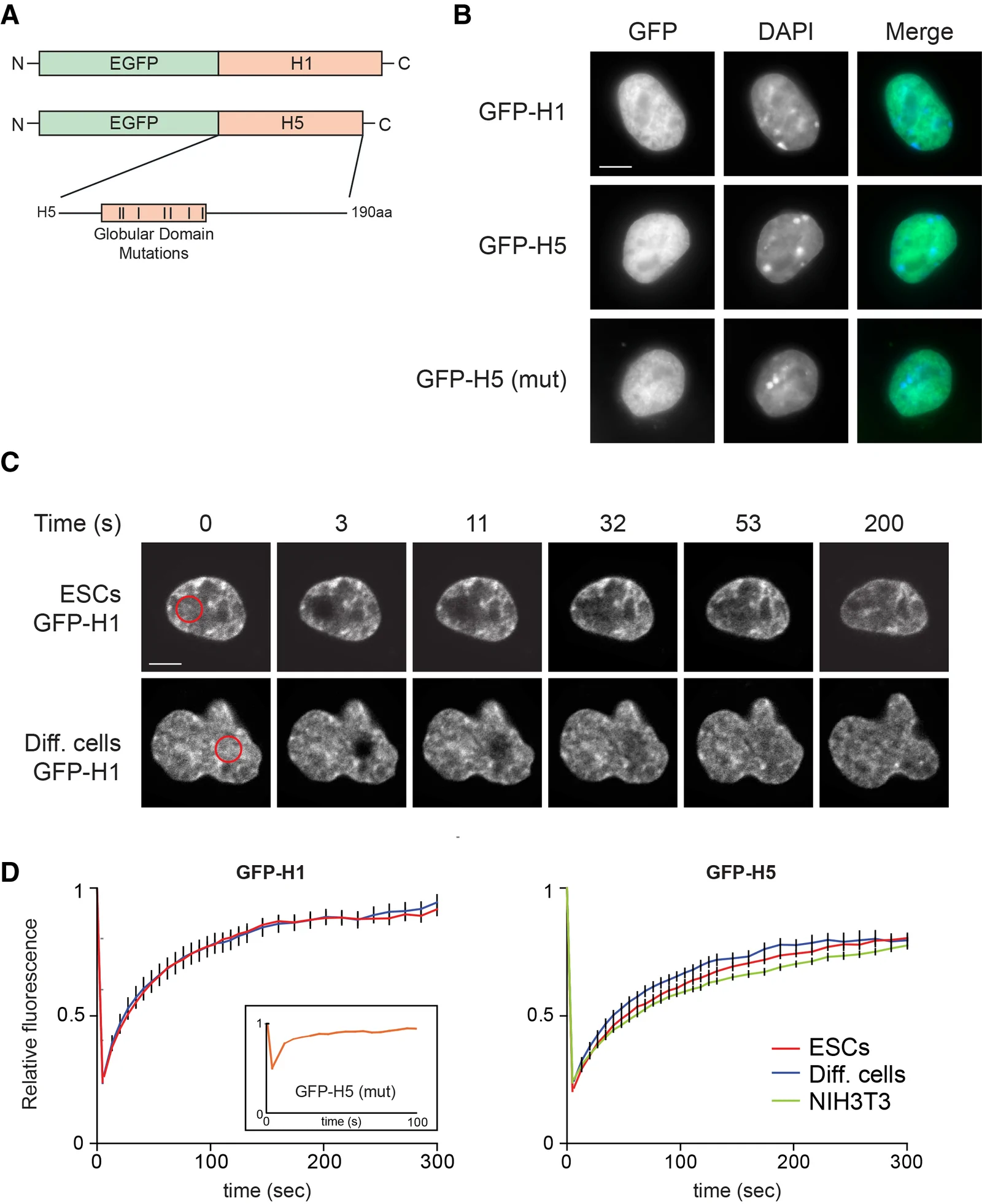
Linker histone mobility in ESCs and differentiated cells (A) Schematic showing linker histones H1 and H5 tagged at their N-termini with EGFP and the position of the globular domain H5 mutations. (B) Distribution of GFP fluorescence (green) in ES cells transfected with GFP-H1, GFP-H5, or GFP-H5 mutants. DNA is counterstained with DAPI (blue). Scale bars, 5 μm. (C) Representative confocal images of GFP-H1-transfected ES cells or differentiated ES cells during FRAP. The bleach area is marked with red circles, and fluorescence recovery is monitored over time. (D) Relative fluorescence within the bleach ROI during FRAP of GFP-H1 (left), GFP-H5 (right), or GFP-H5 mutants (inset) expressed in ES cells (red), differentiated ES cells (blue), or NIH3T3 cells (green). Graphs show median values (±SEM, error bars). 3T3 H5, *n* = 21; ES H1, *n* = 32; Diff H1, *n* = 43; ES H5, *n* = 32; Diff H5, *n* = 41.

### Characterization of *in vivo* fixed stem cell and differentiated cell chromatin

The studies described so far have compared the structure of unfixed chromatin fibers isolated from NIH3T3 cells, ESCs, and differentiated cells (Figure 4) using sucrose gradient sedimentation. While evidence suggests that these buffer conditions closely mimic the nuclear environment^34^^,^^55^ and accurately preserve the structure of higher-order chromatin fibers, there remains a possibility that isolating chromatin fibers alters their conformation, potentially obscuring structural differences. To address this limitation, we developed an approach to investigate chromatin fibers cross-linked within cells to preserve their native structure; we refer to this method as SPOCC.

To evaluate SPOCC, we performed a series of control experiments. First, formaldehyde cross-linking was evaluated to ensure that it would maintain the structure of the higher-order chromatin fibers (Figure S2A). Chromatin fibers were prepared from NIH3T3 cells and dialyzed into either low-salt (1 mM) or high-salt (80 mM) conditions to alter their structure. These fibers were extensively cross-linked with 1% formaldehyde and then dialyzed into a buffer containing 1 mM NaCl/Tris to quench and remove any residual formaldehyde. In the absence of cross-linking, low-salt conditions would be expected to unfold chromatin fibers. After sedimentation, chromatin fibers were isolated by fractionation, cross-links were reversed, and DNA was purified and examined by agarose gel electrophoresis (Figure S2B). By plotting the relationship between DNA size and fraction number (Figure S2C), we showed that despite sedimentation in low-salt buffer, chromatin fibers cross-linked in 80 mM NaCl sedimented more rapidly (compare 6 kb fibers in Figure S3C; 1 mM fibers sedimented in fraction 13, whereas 80 mM fibers sedimented in fraction 16). This result demonstrates that extensive formaldehyde cross-linking effectively preserves distinct chromatin fiber structures.

Second, formaldehyde cross-linking generates extensive intra- and inter-chromatin fiber cross-links, substantially reducing the ability to release chromatin fibers from cells. To address this, we hypothesized that digesting histone tails with trypsin after extensive cross-linking could facilitate chromatin fiber release. Soluble chromatin was isolated from NIH3T3 cells, dialyzed into low- and high-salt buffers, and extensively cross-linked using formaldehyde. The chromatin was then dialyzed to remove formaldehyde and lightly digested with trypsin (Figure S3A), resulting in the clipping of histone tails (Figure S3B). Furthermore, cross-linking was similarly efficient on unfolded or folded chromatin fibers, as evidenced by the appearance of new protein bands in the 1 mM and 80 mM samples, while the protease digested the histones to their trypsin-resistant core.^69^ Chromatin fibers were solubilized in SDS and fractionated on sucrose gradients prepared in 1 mM salt. After sedimentation, gradients were fractionated (Figure S3C), cross-links were reversed, and DNA was purified and analyzed by agarose gel electrophoresis (Figure S3D). Analysis of the sedimentation rates for the chromatin fibers (Figure S4E) indicated that the combination of cross-linking and subsequent trypsin digestion maintained detectable differences in chromatin fiber structure.

As our pilot experiments suggested that a combination of cross-linking and trypsin digestion maintained the structural characteristics of chromatin, we decided to use the SPOCC methodology to characterize the properties of cellular chromatin. To explore the reproducibility of the protocol, two samples of NIH3T3 cells were analyzed in parallel. Nuclei were digested with micrococcal nuclease and then extensively cross-linked using formaldehyde. After quenching with Tris and dialysis to remove residual cross-linker, the samples were digested with trypsin, and soluble chromatin was released in the presence of SDS and fractionated on a sucrose gradient. Fractions were collected, and DNA purified and analyzed by agarose gel electrophoresis (Figure S4A). The distribution of the DNA sizes from pairs of fractions was compared (Figure S4B), and the peak DNA size was determined for each fraction. After plotting fraction number (sedimentation rate) against peak DNA size (Figure S4C), the two samples, as expected, were found to have very similar sedimentation properties, showing the reproducibility of the method for two independent samples.

SPOCC was then used to compare ESC and NIH3T3 cell chromatin. Cross-linked and trypsin-digested chromatin was prepared from ESCs and NIH3T3 cells, and the histone proteins were examined by SDS-PAGE to ensure that they were similarly digested (Figure 6B). The soluble chromatin was fractionated based on its size and structure on a sucrose gradient, and DNA from individual fractions was analyzed by agarose gel electrophoresis (Figure 6C). The distributions of DNA sizes from individual fractions were compared (Figures 6D and 6E) and indicated that ESC chromatin was marginally more disrupted than NIH3T3 chromatin, consistent with the nuclease sensitivity observed in Figure 4F. These results suggest that SPOCC can discriminate between different chromatin structures but also show that some aspects of chromatin folding are lost if the samples are not cross-linked before analysis (Figure 4A). It is well known that some regions of the genome are more heterochromatic than other transcriptionally active regions.^70^ Similarly, polycomb-enriched regions are known to exhibit characteristics of heterochromatin and may be more difficult to extract from cells. To ensure that the chromatin released for analysis was representative of the genome, purified soluble chromatin was fragmented using MNase and analyzed by genome-wide next-generation sequencing. The chromatin released was evenly distributed across the genome (Figure S5A). To further characterize the distribution of released chromtin, we used the chromHMM classification (Figure S5B). Compared with genomic DNA isolated from B-cells, there was enrichment in more disrupted portions of the genome, such as enhancers and transcribed regions, but there was no obvious depletion in specific regions of the genome that might confound subsequent analysis.

**Figure 6:**
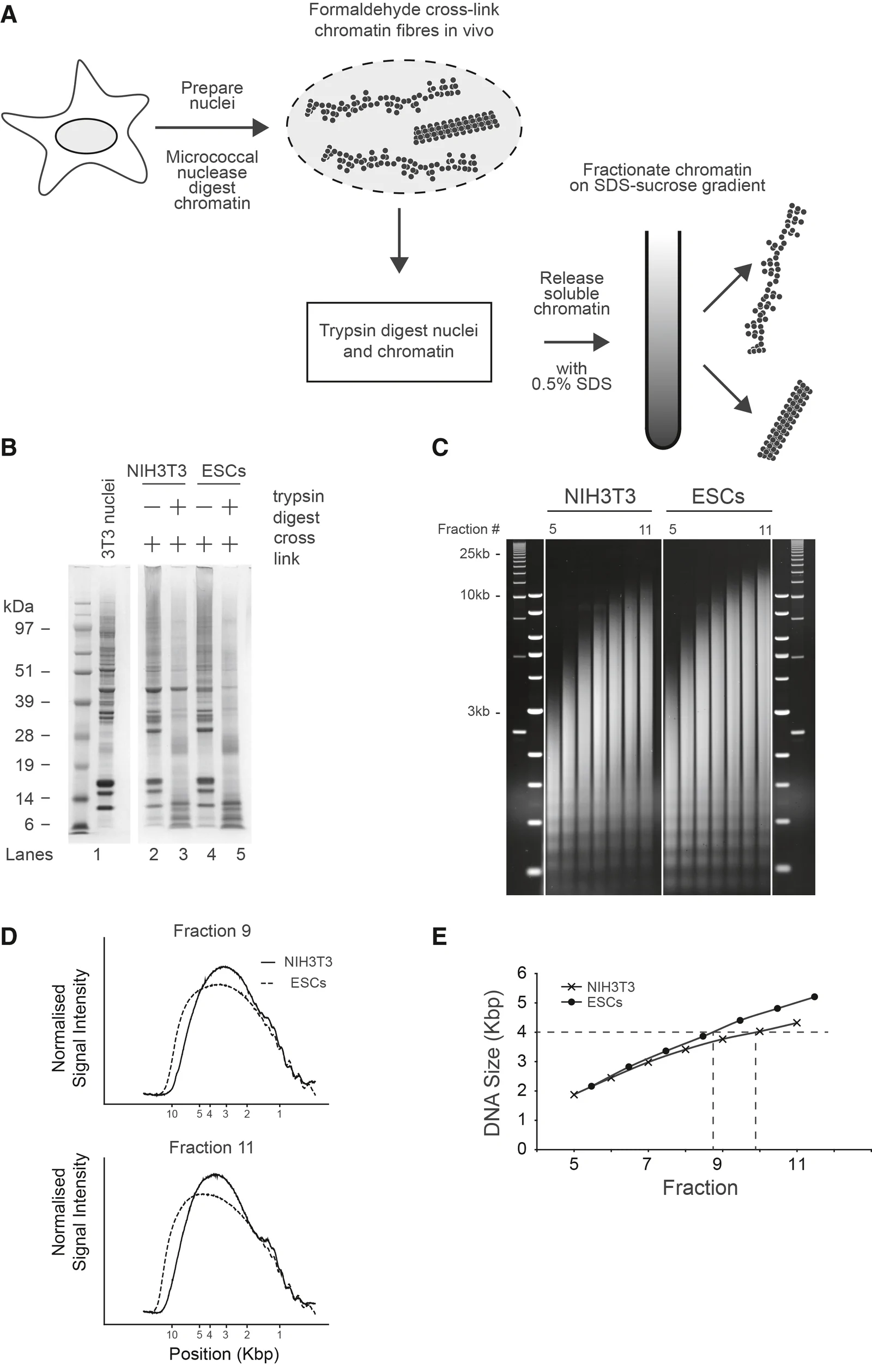
Sedimentation characteristics of chromatin fibers isolated from NIH3T3 and ES cells (A) Schematic showing the preparation, isolation, and characterization of chromatin fibers cross-linked inside cells. Nuclei are digested with MNase and then cross-linked extensively with formaldehyde to maintain intra-fiber cross-links. To isolate the chromatin, inter-fiber cross-links are broken by digestion with trypsin, and the chromatin is solubilized and fractionated based on its (fixed) structure in a sucrose gradient. (B) Qualitative analysis of nuclear proteins isolated from cross-linked NIH3T3 and ES cells in the presence or absence of trypsin. The gel was stained with Coomassie brilliant blue. (C) DNA isolated from individual sucrose gradient fractions analyzed on an agarose gel, stained with ethidium bromide, and imaged using a laser scanner. (D) Densitometry of fraction 14 (top) and fraction 16 (bottom) for NIH3T3 and ESC DNA. (E) Graph showing the relationship between DNA size and fraction (sedimentation rate), determined from the gel shown in (C). There were two independent experiments; samples were run on multiple gradients and analyzed on multiple gels. Representative results are shown here. See also Figures S2–S5.

As ESCs and NIH3T3 cells are very different cell types, we decided to directly compare stem cells and differentiated stem cells using our SPOCC method. Nuclei were prepared from ESCs and differentiated cells, digested with micrococcal nuclease, and cross-linked. The chromatin was solubilized and fractionated on a sucrose gradient (Figure 7A). Subsequently, DNA was isolated from individual fractions and analyzed by agarose gel electrophoresis (Figure 7B). Analysis of individual fractions (Figure 7C) and all fractions indicated that chromatin fibers from stem cells and differentiated cells sedimented at the same rate, indicating that they have very similar bulk chromatin fiber structures.

**Figure 7:**
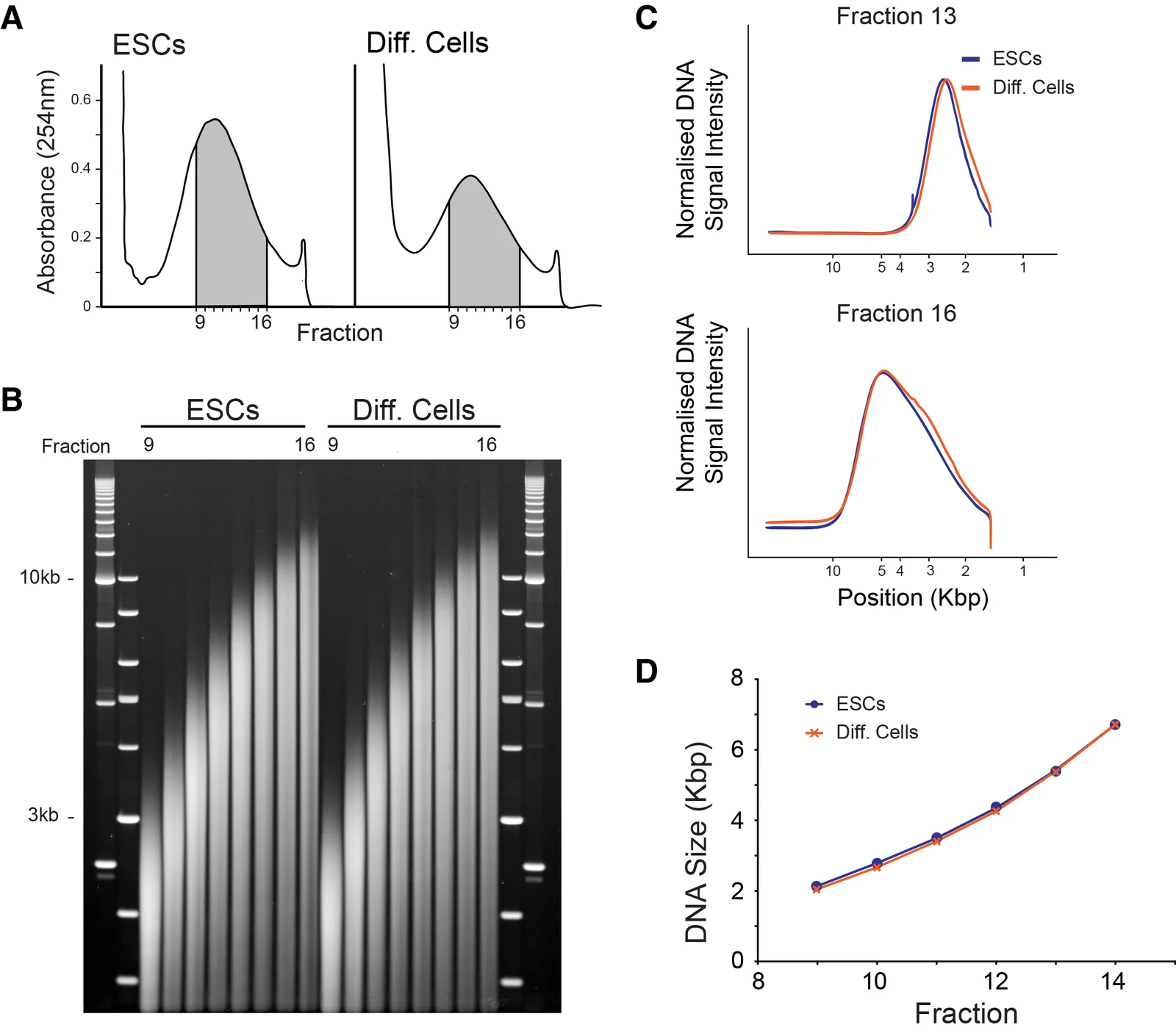
Sedimentation properties of stem cell and differentiated cell chromatin fibers cross-linked inside nuclei (A) Cross-linked chromatin isolated from ES cells and differentiated cells and fractionated in an isokinetic sucrose gradient in the same centrifuge in the presence of 1 mM NaCl. Graphs showing the absorbance (254 nm) across the sucrose gradients (top of gradient is on the left side). (B) DNA purified from individual fractions from each gradient (gray area in A) fractionated on an agarose gel, stained with ethidium bromide, and scanned using a laser scanner. (C) Densitometry of ethidium bromide staining in individual lanes shown in panel (C). (D) Relationship between DNA size and sedimentation rate (fraction) for stem cell and differentiated cell chromatin. Four independent experiments were performed. Samples were analyzed on multiple gradients and multiple gels. Representative results are shown in the figure.

## Discussion

Our study indicates that bulk chromatin fibers isolated from stem cells and differentiated stem cells have equivalent structures (Figure 7D), contrary to the accepted dogma, and instead suggests that the organization of the higher-order chromatin fiber does not contribute to the pluripotent stem cell state, *per se*. Synthetic disrupted chromatin fibers form “puddles,”^17^ that, by super-resolution imaging in cells, appear to look like “clutches.”^71^ Pluripotent cells have less dense clutches containing fewer nucleosomes than differentiated cells, suggesting that this difference might be linked to the stem cell state. However, the hydrodynamic sedimentation approaches used in this study failed to detect differences in the conformation of the higher-order chromatin fiber between bulk chromatin from stem cells and differentiated cells. This is unlikely to be due to a limitation in the technique, as this approach can detect small differences in chromatin fiber folding in the vicinity of promoters in transcriptionally active genes,^31^ and we were able to observe that ESCs had a slightly more disrupted bulk chromatin fiber structure than NIH3T3 cells (Figure 6). Although these differences are difficult to quantify, they are indicative of additional disruptions in the chromatin fiber. Furthermore, when chromatin was analyzed under conditions favorable to higher-order folding, satellite-containing heterochromatin had a sedimentation rate approximately 20% greater than of bulk chromatin.^54^ In this study, we were unable to find any substantive evidence for differences in bulk chromatin between stem cells and differentiated cells; therefore, differences in the conformation of higher-order chromatin fibers may only be in specific regions of the genome, such as satellite-containing centromeric heterochromatin,^54^^,^^72^^,^^73^ localized regions around transcription start sites,^31^ or transcriptionally active genes.^74^ In these situations, nucleosomes are dynamically remodeled to facilitate access to transcription-factor-binding sites and docking of RNA polymerase. These regions are nuclease-sensitive and readily degraded by DNase I, revealing regions that are often referred to as hypersensitive sites. Mapping studies suggest that most active genes have regions of disrupted chromatin covering approximately 150–200 bp. These regions are unlikely to be detected in this study for two reasons. First, they are too small to have a significant effect on the sedimentation of chromatin fibers. Second, because these regions are sensitive to nucleases, they are likely to be degraded by MNase early in the digestion process. Although chromatin remodeling machines like Chd1 play a role in maintaining stem cell genes in a special “open” state,^10^^,^^46^ remodeling enzymes are unlikely to have a global effect on chromatin structure. Our results are consistent with stem cell and differentiated cells having broadly similar levels of chromatin fiber compaction.

Previously, we estimated the extent of differences in chromatin fiber structure between bulk chromatin, satellite-containing chromatin,^54^ and chromatin observed at promoters.^31^ It would similarly be interesting to determine the molecular basis for differences in bulk chromatin structure between NIH3T3 cells and ESCs (Figure 6). To do this, however, it would be necessary to map different chromatin structures at higher resolution using, for example, next-generation sequencing. This would indicate whether the differences observed in these experiments are limited to specific genomic regions or are more global. Although the basis for this difference is not known, one possibility is that, as ESCs replicate more rapidly than NIH3T3 cells, their chromatin does not fold into such a regular structure. It is well known that H1.0 is expressed in terminally differentiated cells,^75^ compacting chromatin^76^ and repressing transcription.^77^ Recent reports suggest that fibroblasts express increased amounts of H1.0, which might have the effect of compacting the chromatin fiber.^76^

Human embryonic stem cells and iPS cells have great therapeutic potential as source of specific cell types for treating diseases. Central to the use of stem cells is our ability to understand the cellular processes required to regulate their differentiation pathways. This study demonstrates that, although stem cells are poised for differentiation along different lineages, their bulk higher-order chromatin fiber is a structure predominantly used for the regular packaging of DNA rather than providing a generally permissive environment for gene expression. Instead, we suggest that the binding of master transcription factors to key regulatory genes is responsible for stemness and is sufficient to impose or re-impose a stem cell state without the need to modify global higher-order chromatin fiber structures.

### Limitations of the study

Sucrose gradient sedimentation has been used by us^31^^,^^54^^,^^55^^,^^59^^,^^78^ and other researchers^79^ to evaluate the structural properties of soluble chromatin fibers. In this approach, soluble chromatin is isolated from cells and then characterized outside of its cellular context. Although this allows questions to be asked about the fundamental principles of chromatin folding, there is a significant possibility that it does not reflect all aspects of chromatin folding inside the nucleus. Not least, many chromatin proteins are lost when chromatin is isolated, supercoiling is also released when chromatin is cut by nucleases, and nuclear processes such as transcription are significantly abrogated. To overcome these limitations when studying cellular chromatin, it would be better to “fix” chromatin within the confines of the nucleus before analysis. Chromatin fixation is straightforward, but the isolation of fixed chromatin is challenging. In this study, we developed SPOCC, a technique that enables chromatin fibers fixed in the nucleus to be isolated and examined. To achieve this, cross-linked nuclei were briefly digested with trypsin to remove histone tails and then solubilized using SDS. Although we provide controls to demonstrate that this method can still discriminate between chromatin fibers with different structures, there is a possibility that other aspects of chromatin folding will also be lost. Notably, fixation and manipulation of the chromatin fiber will affect chromatin fiber dynamics. Similarly, fixation might work with different efficiencies in different parts of the nucleus. It is therefore important to interpret results using SPOCC with an understanding of its potential limitations and to complement them, where possible, with other experimental techniques.

## Resource availability

### Lead contact

Requests for further information and resources should be directed to and will be fulfilled by the lead contact, Nick Gilbert (nick.gilbert@ed.ac.uk).

### Materials availability

This study did not generate new unique reagents.

### Data and code availability

• Chromatin release data (Figure S5) have been deposited at GEO (Gene Expression Omnibus) under accession number GSE346906 and are publicly available as of the date of publication.
• This paper does not report original code
• Any additional information required to reanalyze the data reported in this paper is available from the lead contact upon request.

## Acknowledgments

The authors would like to thank members of our research groups for useful discussions and Dirk-Jan Kleinjan and Catherine Naughton for commenting on the manuscript. We also acknowledge Matt Pearson of the Advanced Imaging Resource at the Institute of Genetics and Cancer for his technical support. This work was supported by the 10.13039/501100000265UK Medical Research Council (MC_UU_00035/6).

## Author contributions

Conceptualization, N.G. and J.A.; methodology, J.D. and N.G.; investigation, J.D., S.B., and N.G.; writing – original draft, N.G. and J.A.; writing – review and editing, J.D., S.B., and N.G.; funding acquisition, N.G.

## Declaration of interests

The authors declare no competing interests.

## STAR★Methods

### Key resources table

**Table:** 

REAGENT or RESOURCE	SOURCE	IDENTIFIER
**Antibodies**		

Rhodamine anti-dig sheep antibody	Roche	11 207 750 910
Texas Red anti-sheep antibody	Vector	TI-6000
Biotinylated anti-avidin antibody	Vector	BA-0300

**Chemicals, peptides, and recombinant proteins**		

DFF/CAD nuclease	Synthesised in lab	N/A
Mouse C_0_T-1 DNA	Roche	N/A
Avidin-FITC	Vector	N/A
Trypsin	Sigma	T8128
Soya bean trypsin inhibitor	Sigma	T9003
NP40	Calbiochem	492015

**Deposited data**		

Chromatin release data	GEO	GSE346906

**Experimental models: Cell lines**		

NIH3T3cells		N/A
OS25 mouse embryonic stem cells	Billon et al.^80^	N/A
ht2 mouse embryonic stem cells	Austin Smith (University of Exeter)	N/A

**Recombinant DNA**		

DFF45 and DFF40 subunits cloned in pRSFDuet	Xiao et al.^60^	N/A
GFP-H1	Gilbert et al.^59^	N/A
GFP-H5	Gilbert et al.^59^	N/A
GFP-H5 mutant	This study	N/A

**Software and algorithms**		

IPLab		N/A
Bowtie2 v2.5.3		N/A
Bedtools v2.31.1		N/A
ChromHMM	https://compbio.mit.edu/ChromHMM/	N/A
NIS Elements AR	Nikon	N/A
Aida v3.22 analysis software	Raytest	N/A

### Experimental model and study participant details

The cell lines used in this study were NIH3T3 mouse embryonic fibroblasts, ht2 (*Oct-hyg-tk*) and OS25 ESCs (*Oct-hyg-tk*, *Sox2βgeo*).^80^^,^^81^^,^^82^ Cell lines were routinely checked for mycoplasma contamination.

### Method details

#### Cell culture and differentiation

NIH3T3cells were grown in high-glucose DMEM supplemented with 10% FCS and 1% pen/strep in 5% CO_2_. ESCs were grown on plastic coated with 0.1% gelatin in GMEM supplemented with 10% FCS, 2 mM glutamine, 100 mM sodium pyruvate, 1% pen/strep, 1% non-essential amino acids, 100 μM 2-ME and 100U/mL LIF in 5% CO_2_. OS25 ESCs were differentiated with retinoic acid (RA) using an abbreviated 5-day protocol.^81^^,^^83^ For this protocol 5 × 10^−6^ cells were plated in a 100 mm dish in complete media and grown overnight. On day 1 media was changed with LIF being replaced by 5 μM RA, and on day 3 fresh media supplemented with RA was added. Cells were harvested on day 5. Gene expression was followed using RT-PCR and expression arrays.^84^

#### Nuclei and chromatin preparation and nuclease digestion

Nuclei and chromatin were prepared using previously optimised methods.^54^^,^^55^ Cell cultures were harvested in PBS containing 0.25 mg/mL trypsin and 1 mM EDTA and were washed in PBS. The cell pellet was resuspended in a small volume of ice-cold NBA (85 mM KCl, 10 mM Tris⋅HCl [pH 7.6], 5.5% (w/v) sucrose, 0.5 mM spermidine, 0.2 mM EDTA, 0.25 mM PMSF). To this an equal volume of NBB (NBA plus 0.05% (v/v) NP-40 (Calbiochem, 492015) was added, and the cells were incubated on ice for 3.5 min. Nuclei were collected by centrifugation (360 × *g* for 3.5 min at 4°C) and washed in NBR (85 mM KCl, 10 mM Tris-HCl [pH 7.6], 5.5% (w/v) sucrose, 1.5 mM CaCl_2_, 3 mM MgCl_2_, 250 μM PMSF) before resuspending at 20 A_260_ units/mL in NBR.

To determine nucleosome repeat length, nuclei were prepared and diluted to 4 A_260_ units/mL in NBR buffer and were digested with 10–40 units/mL Mnase or 10–40 μL/mL DFF/CAD nuclease.^60^ Aliquots were removed into stop buffer at suitable times and DNA was purified and analyzed on 1.2% agarose gels in TBE buffer. Ethidium bromide stained agarose gels were scanned using a 532 nm laser and a 580 nm band-pass filter on a Fuji FLA-5000. The size of the bands was determined from the DNA size markers using Aida v3.22 analysis software. Nucleosomal bands were accurately measured for each time point and the data were plotted as band number against fragment size. The gradient of this relationship provides the nucleosome repeat length for each time point of digestion. The repeat length was taken as the cross-over point for digests undertaken with different concentrations on DFF/CAD nuclease.

To prepare soluble chromatin, nuclei were diluted to 20 A_260_ units/mL in NBR buffer and digested with 6–10 units/mL MNase. Digestion was stopped with 10 mM EDTA and the nuclei were resuspended in TEEP20N buffer (10 mM Tris-HCl pH 8.0, 1 mM EDTA, 1 mM EGTA, 250 μM PMSF, 20 mM NaCl, 0.05% NP40) overnight. For chromatin purification nuclear debris was removed by 5 min centrifugation in a microfuge and 850 μL soluble chromatin was layered on a 10%/50% sucrose step gradient in TEEP80 buffer (10 mM Tris-HCl pH 8.0, 10 mM EDTA, 1 mM EGTA, 250 μM PMSF, 80 mM NaCl) and centrifuged at 4°C (50,000 rpm) for 1h 50 min in an MLS-50 rotor in a benchtop ultracentrifuge (Beckman). For chromatin structure analysis nuclear debris was removed by centrifugation and 400 μL soluble chromatin was loaded on to a 6–40% isokinetic sucrose gradient and centrifuged at 4°C (41,000 rpm) for 3.5 h s in an SW41 rotor in TEEP80 buffer. 500 μL fractions were collected from the gradients by upward displacement. DNA was purified from chromatin by phenol-chloroform extraction whilst proteins were purified by ethanol precipitation or extraction with Tri-Reagent (Sigma) followed by ethanol precipitation. Proteins were analyzed on 12% NuPAGE gels (Invitrogen).

#### Cesium chloride density centrifugation

Soluble chromatin was purified on a 10%/50% step gradient as described above. The peak chromatin fraction (500 μL) was equilibrated into TEAP80 buffer (10 mM Triethanolamine-HCl pH 8, 1 mM EDTA, 80 mM NaCl, PMSF) using a MiniTrap Sephadex G-25 spin column (Cytiva), supplemented with 0.5% formaldehyde and rotated overnight at 4°C. The density of the samples were adjusted to 1.42 g/mL by adding 0.8 vol. saturated cesium chloride (1.92 g/mL) and centrifuged in a Sorval TV-865 rotor at 20°C (55,000 rpm) for at least 40 h. 250 μL fractions were collected from the bottom of the isopycnic gradients, refractive index (RI) was measured using a refractometer (Bellingham Stanley) and the DNA concentration was determined by measuring A_260_. Cesium chloride density = 10.819 × RI – 13.441.

#### Histone H1 and H5 constructs

Plasmids for GFP-H1 and GFP-H5 were described previously.^59^ To make a GFP-H5 binding mutant positively charged lysine and arginine amino acids in the H5 globular domain were replaced with either alanine or glutamic acid (K40E, R42E, K52A, K69A, R73A, K85A, R94A),^65^ the globular domain was then cloned into full length H5, and fused to GFP.

#### FRAP

ESCs and differentiated daughter cells were grown on 35 mm glass bottom dishes (Iwaki) and transfected with either GFP-H1, GFP-H5 or a GFP-H5 binding mutant. 24 h after transfection, samples were mounted onto a Nikon A1R, equipped with a Solent Scientific incubation chamber incorporating temperature and humidified CO_2_ control. The microscope comprised of a Nikon Eclipse TiE inverted microscope with Perfect Focus System and was equipped with a 457/488/514 nm Multiline Argon lasers. Data were acquired using NIS Elements AR software (Nikon Instruments Europe, Netherlands).

Cells expressing high levels of H1- or H5-GFP fusion protein (total cellular pixel intensity > 35,000) were excluded from analysis. For FRAP, a 4-μm-diameter region of interest (ROI) of the nucleus in the midfocal plane was bleached for 3 s using the 488 laser (30%) power. Images were captured with a 60× objective at 7-s intervals for a total of 120 s and then 14 s intervals for 180 s using 10% of laser power. Each image was processed by an interactive script (IPLAB version 3.6; Scanalytics) to correct for nuclear rotation and cell movement. Loss of fluorescence attributed to the imaging process alone was assessed from the sum of pixel intensities in the cell. The fluorescence intensity for each ROI over time was then normalized to this.

#### FISH

To examine the representative release of soluble chromatin (Figures S2D and S2E), DNA purified from chromatin fractions was labeled with either biotin or digoxigenin and hybridised to 3:1 fixed metaphase spreads prepared from NHI3T3 cells. Mouse C_0_T-1 DNA was included in hybridisations to suppress signal from repetitive DNA. Biotinylated probes were detected using sequential layers of avidin-FITC and biotinylated anti-avidin. Digoxigenin-labeled probes were detected using rhodamine anti-dig sheep antibody and Texas Red-*anti*-sheep antibody (Vector). Slides were counterstained with 0.5 μg/mL DAPI Slides were examined with an epifluorescence microscope (Axioskop; Carl Zeiss MicroImaging, Inc.) equipped with a 100× NA 1.3 lens and a CCD camera (Micromax; Princeton Instruments).

#### SPOCC

To examine the Sedimentation Properties of Cross-Linked Chromatin (SPOCC), nuclei were prepared and micrococcal nuclease digested as described above. Nuclei were washed in TEAP80 buffer and cross-linked in 1% formaldehyde 10 min at RT. The sample was boosted to give 2% formaldehyde and cross-linked at 4°C for 4 h on a wheel. Samples were dialyzed against TEN20P (10 mM Tris-HCl pH 7.5, 1 mM EDTA, 20 mM NaCl, PMSF) overnight and digested with 40 μg/mL trypsin (Sigma T8128, 1500 units/mg). The reaction was stopped by adding 40 μg/mL soyabean trypsin inhibitor (Sigma T9003), PMSF and SDS to 0.5%. Samples were incubated 10 min at room temperature and then loaded on to a 6–40% isokinetic sucrose gradient and centrifuged at 4°C (41,000 rpm) for 4.5 h s in an SW41 rotor in TEEP1 buffer (10 mM Tris-HCl pH 8.0, 10 mM EDTA, 1 mM EGTA, 250 μM PMSF, 1 mM NaCl). 500 μL fractions were collected from the gradients by upward displacement and the DNA from them was purified by phenol-chloroform extraction and analyzed by gel electrophoresis.

#### *In vitro* chromatin cross-linking and trypsin digestion

Soluble chromatin was prepared as described above and dialyzed against TEAP1 (10 mM Triethanolamine-HCl pH 8, 1 mM EDTA, 1 mM NaCl, PMSF) or TEAP80 buffer. Chromatin in 1 mM NaCl adopts an unfolded, 10-nm, configuration whilst in 80 mM NaCl it is folded to give a 30-nm fiber.^34^ To cross-link the samples formaldehyde was added to 1% and incubated at 4°C for 6 h on a wheel. Samples were then processed as for *in vivo* chromatin cross-linking and digestion.

#### DFF/CAD nuclease preparation

Engineered mouse DFF45 and DFF40 subunits were cloned in pRSFDuet (Novagen) (gift from W.Garrard, University of Texas Southwestern Medical Centre)^60^ to enable co-expression. The plasmid was transformed into BL21(DE3)-RP cells (Stratagene). Cells were grown until 2OD and then induced using 1 mM IPTG at 16°C. After 12 h cells were harvested and lysed in Binding buffer (300 mM NaCl, 15 mM Imidazole, 50 mM Tris-HCl (pH 8), 10% Glycerol, 10 mM 2 ME, PMSF, Protease inhibitor tablet). Lysozyme was added to 1 mg/mL for 30 min on ice and the cells were extensively sonicated. The mixture was clarified by adding Triton X-100 to 0.1% and cleared by centrifugation. The protein was then purified on nickel agarose using standard techniques, desalted into storage buffer (100 mM KCl, 20 mM Tris-HCl pH 8.0, 0.2 mM EDTA, 2 mM DTT, 10% Glycerol) and diluted to give 50% glycerol. Protein quality was estimated by gel electrophoresis and activity was characterised by digesting naked DNA and nuclei. The DFF/CAD nuclease was activated by digestion with TEV enzyme (Invitrogen) at 30°C and was used in NBR buffer (5.5% sucrose, 85 mM KCl, 10 mM Tris-HCl pH 7.5, 1.5 mM CaCl_2_, 3 mM MgCl_2_, PMSF).

#### Nuclease digestion of purified chromatin

A single isokinetic fraction of soluble chromatin purified by sedimentation in a 6–40% isokinetic sucrose gradient was supplemented with 3 mM CaCl_2_ and digested with micrococcal nuclease (0.2 units/μg) at room temperature. An 80 μL aliquot was removed into MNase stop buffer (1% SDS, 100 μg/mL proteinase K, 2.5 mM EDTA) for time 0. To the remaining material, micrococcal nuclease was added and 80 μL aliquots were removed on a time course basis into MNase stop buffer. The DNA was purified for agarose gel electrophoresis. All the chromatin samples tested negative for endogenous nucleases or residual micrococcal nuclease within the time frame of these experiments.

#### Agarose gel electrophoresis and analysis

Large DNA fragments were fractionated by gel electrophoresis in 0.7–1% agarose in 1× TPE buffer (90 mM Tris-phosphate, 2 mM EDTA) with buffer circulation whilst small DNA fragments were fractionated by gel electrophoresis in 1%–1.2% agarose in 1× TBE buffer (50 mM Tris-borate, 1 mM EDTA). Ethidium bromide stained agarose gels were scanned using a 532 nm laser and a 580 nm band-pass filter on a Fuji FLA-5000. The size of the bands was determined from the DNA size markers which were a 2.5 kb DNA ladder (Bio-Rad) or a 1 kb or 100 bp DNA ladder (NEB) using Aida v3.22 analysis software.

#### Analysis of chromatin genome-wide coverage

Soluble chromatin from mouse ES cells and differentiated cells was prepared as described above. DNA was extracted using SDS, proteinase K digestion, phenol:chloroform:isoamyl-alcohol extraction, and ethanol precipitation. DNA was electrophoresed on a 0.7% TPE gel for 24 Vh/cm and then extracted from a gel slice using beta agarase (NEB), focusing on 2–2.5 kb modal molecular weight DNA. This was used to generate libraries using the NEBNext® Ultra™ II FS DNA Library Prep, with minimal amplification (8 cycles). Sequencing was performed on illumina NextSeq P3 flow cell (Novogene), generating 50 bp paired end reads.

Alignment of reads to GRcm38 was performed using Bowtie2 (v2.5.3, –very-sensitive-local), followed by filtering to remove alignments with a MAPQ < 20. The mean depth of properly paired primary alignments was calculated for each 1 kb window of GRCm38 using Bedtools coverage (v2.31.1). This was then normalised based on the number of filtered alignments, read length, and effective genome size (depth per genomic coverage, DPGC). Smoothing was performed, per chromosome, using a 1 Mb rolling median. A similar approach was taken to calculate the DPGC in chromatin states generated using E14 ChIP-seq data.^85^

For comparison PCR free whole genome sequencing data from three mouse strains^86^ was analyzed using the same approach. Down sampling was performed to retain a random selection of 10% of reads.

### Quantification and statistical analysis

For analysing nucleosome repeat length the size of individual bands was accurately determined and plotted in Excel. Curves were fitted to a logarithmic trendline. The repeat length was taken as the cross-over point for digests undertaken with different concentrations of enzyme, and was calculated by solving the two logarithmic trendlines. Samples were digested in two independent experiments with different concentrations of enzyme and analyzed on four different gels, giving four different calculated repeat lengths. The median and standard deviation were calculated in Excel. For FRAP data from individual cells were independently analyzed. For each time point the median and standard error of the mean were calculated in Excel. For next generation sequencing analysis DPGC (depth of genomic coverage) was provided by the chromHMM classifier. Medians were calculated in python and boxplots were generated in python. Lower and upper limits of boxes correspond to the first and third quartile, with the mid-point of the box being the median. By standard convention whiskers are 1.5 × the inter-quartile range (IQR). Further information and values of n are shown in figure legends.
