# Supplementary material for: Embryonic stem cells do not have a globally disrupted higher-order chromatin fiber structure
Source: iScience. 2026 Nov 5;29(11):118053. doi: 10.1016/j.isci.2026.118053 (PMC13753350; doi:10.1016/j.isci.2026.118053)

iScience, Volume *29*

Supplemental information

Embryonic stem cells do not have a globally disrupted higher-order chromatin fiber structure

James Ding, Shelagh Boyle, James Allan, and Nick Gilbert

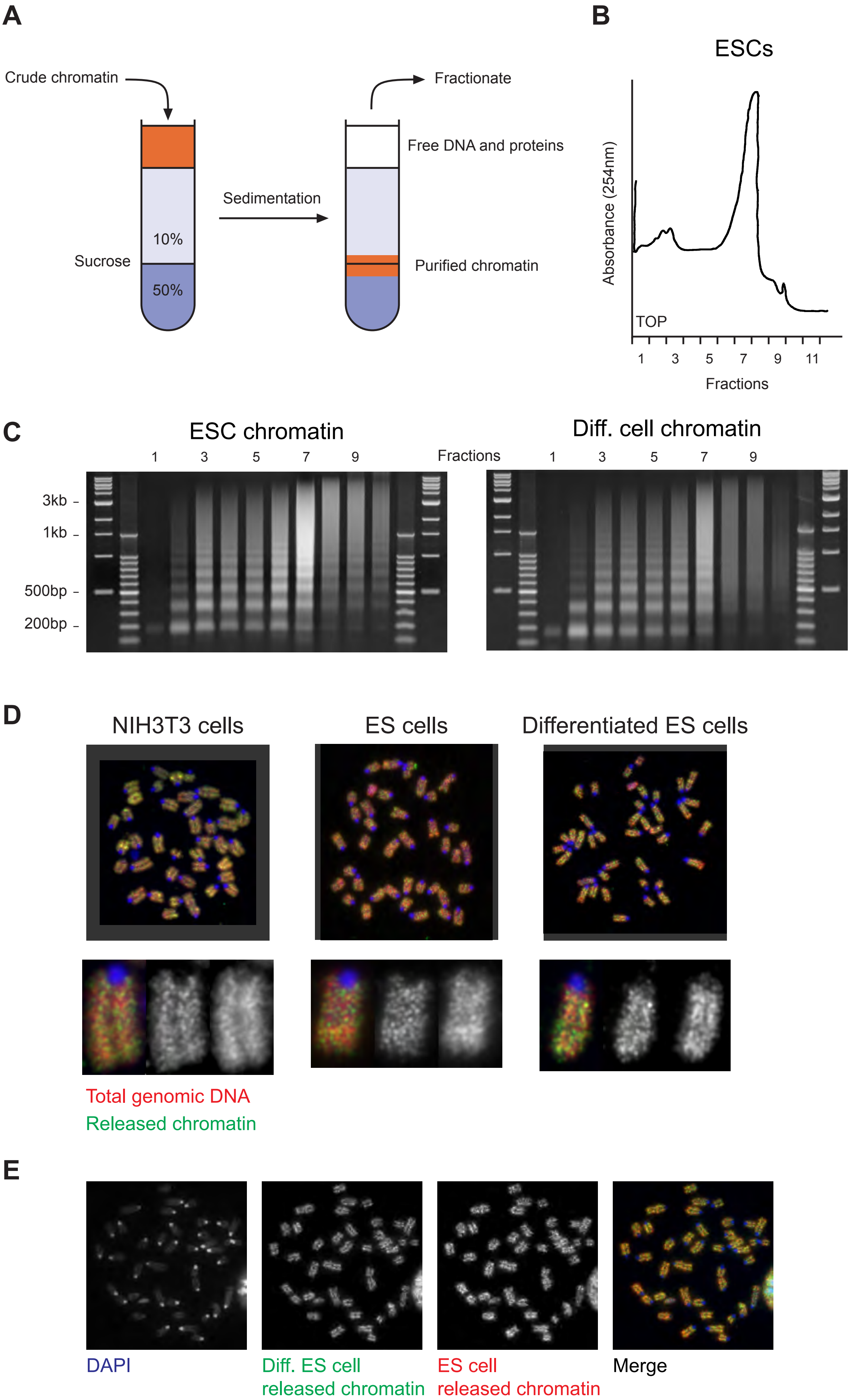
A
Crude chromatin
Fractionate
Free DNA and proteins
Sedimentation
10%
Sucrose
50%
Purified chromatin
B
ESCs
Absorbance (254nm)
TOP
1 3 5 7 9 11
Fractions
C
ESC chromatin
1 3 5 7 9
Fractions
Diff. cell chromatin
1 3 5 7 9
3kb
1kb
500bp
200bp
D
NIH3T3 cells
ES cells
Differentiated ES cells
Total genomic DNA
Released chromatin
E
DAPI
Diff. ES cell released chromatin
ES cell released chromatin
Merge

Figure S1

Figure S1 (related to Figure 2)

Soluble chromatin fibres released from somatic cells, stems cells and differentiated stem cells are representative of the entire genome.

A. Soluble chromatin was isolated from cells and purified on a 10%/50% sucrose step gradient. Fractions were collected by upward displacement.

B. Representative graph showing the absorbance (254 nm) of chromatin across a sucrose gradient (top of gradient is left side).

C. DNA was purified from individual gradient fractions and analysed by gel electrophoresis and stained with ethidium bromide.

D. Peak DNA fractions were taken and labelled by nick translation. Top, DNA isolated from soluble chromatin (green) was comparatively hybridised on a mouse metaphase spread with total genomic DNA (red) from the same cells in the presence of Cot-1. Bottom, enlarged images of selected chromosomes. Chromosomes were counterstained with DAPI (blue).

E. DNA isolated from ES cell soluble chromatin (red) was comparatively hybridised to a mouse metaphase spread with DNA isolated from differentiated ES cell soluble chromatin (green) in the presence of Cot-1. Chromosomes were counterstained with DAPI (blue).

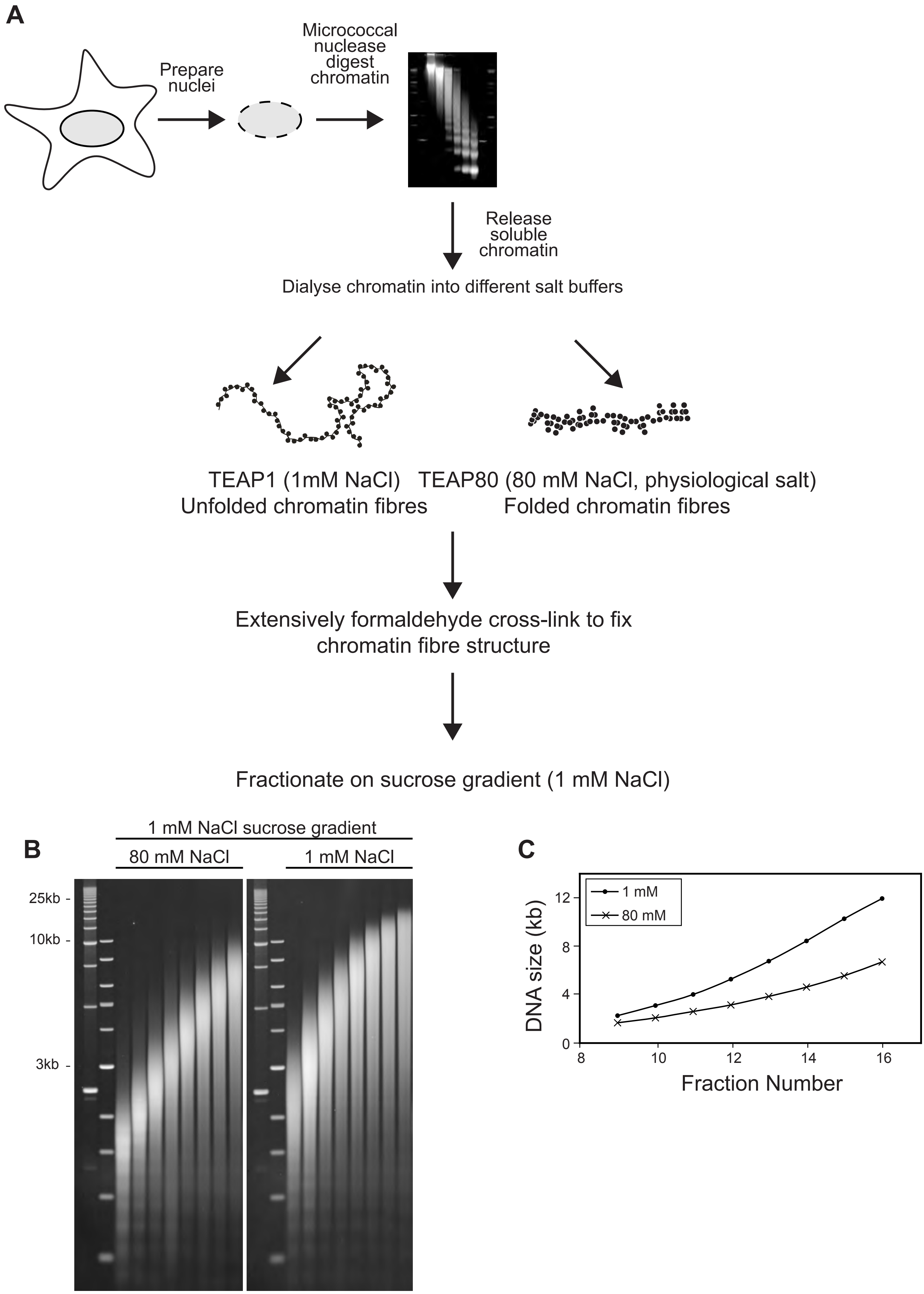
A
Prepare nuclei
Micrococcal nuclease digest chromatin
Release soluble chromatin
Dialyse chromatin into different salt buffers
TEAP1 (1mM NaCl)
Unfolded chromatin fibres
TEAP80 (80 mM NaCl, physiological salt)
Folded chromatin fibres
Extensively formaldehyde cross-link to fix chromatin fibre structure
Fractionate on sucrose gradient (1 mM NaCl)
B
1 mM NaCl sucrose gradient
80 mM NaCl
1 mM NaCl
25kb
10kb
3kb
C
1 mM
80 mM
DNA size (kb)
Fraction Number
0
4
8
12
8
10
12
14
16

Figure S2

Figure S2 (related to Figure 6)
Differently folded and cross-linked chromatin fibres exhibit a pronounced difference in sedimentation rate.

A. Schematic showing the experimental approach. Soluble chromatin fibres were isolated from NIH3T3 cells and either folded in 80 mM NaCl or unfolded in 1 mM NaCl. Chromatin structure was fixed by extensive formaldehyde cross-linking introducing intramolecular bonds. After cross-linking chromatin samples were fractionated on a sucrose gradient in low salt (1 mM NaCl).

B. Chromatin isolated from NIH3T3 cells, structure altered by changing salt concentration, fixed and fractionated in an isokinetic sucrose gradient. DNA was purified from individual fractions from each gradient, cross-links reversed, DNA purified and fractionated on an agarose gel, stained with ethidium bromide and scanned on a Fuji FLA-5000 laser scanner.

C. Graph showing the relationship between DNA size and fraction number (sedimentation rate) for differently folded and cross-linked chromatin fibres.

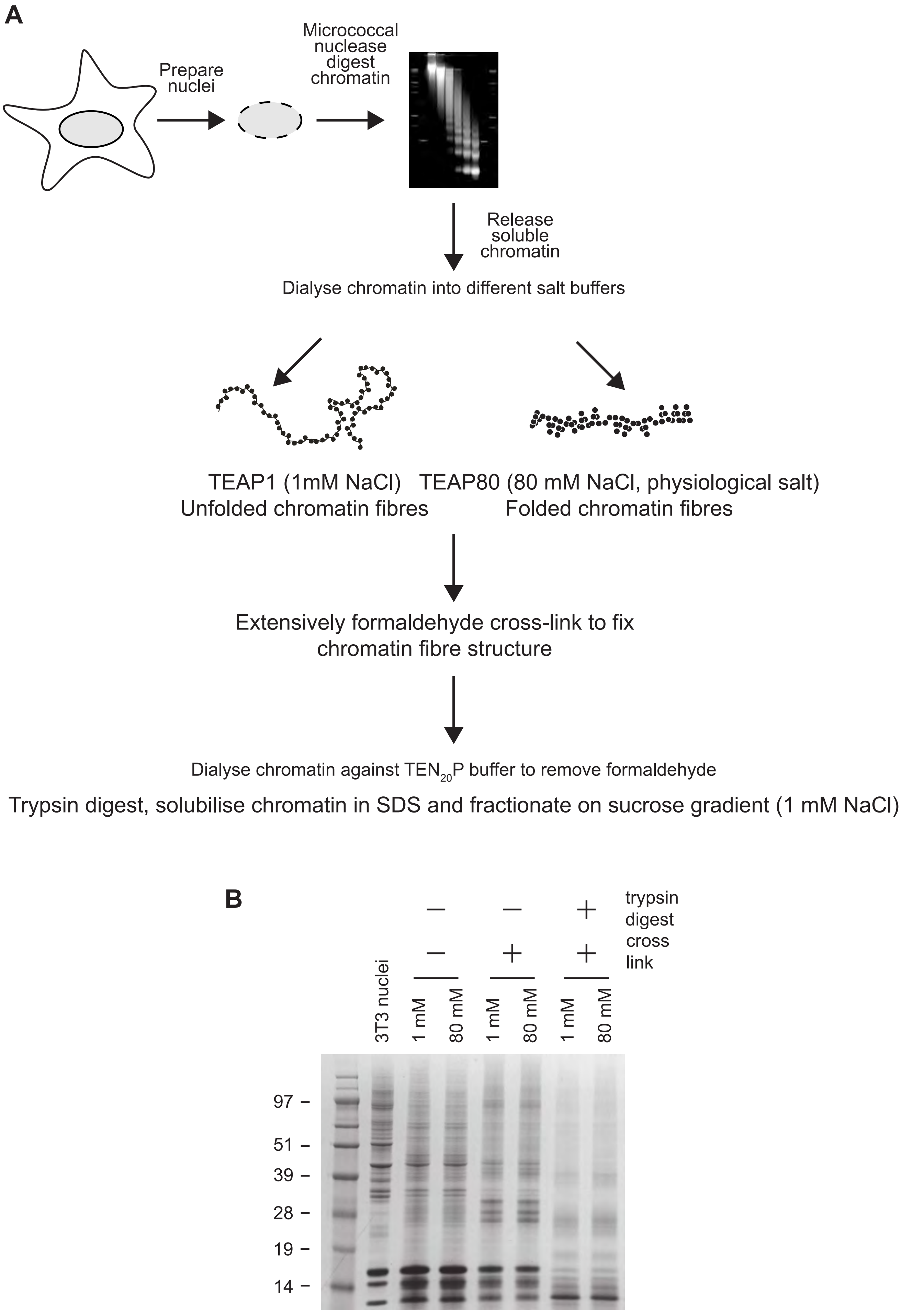

A
Prepare nuclei
Micrococcal nuclease digest chromatin
Release soluble chromatin
Dialyse chromatin into different salt buffers
TEAP1 (1mM NaCl)
Unfolded chromatin fibres
TEAP80 (80 mM NaCl, physiological salt)
Folded chromatin fibres
Extensively formaldehyde cross-link to fix chromatin fibre structure
Dialyse chromatin against TEN$_{20}$P buffer to remove formaldehyde
Trypsin digest, solubilise chromatin in SDS and fractionate on sucrose gradient (1 mM NaCl)
B
trypsin digest
cross link
3T3 nuclei
1 mM
80 mM
1 mM
80 mM
1 mM
80 mM
97
51
39
28
19
14
Lanes 1 2 3 4 5 6 7

Figure S3

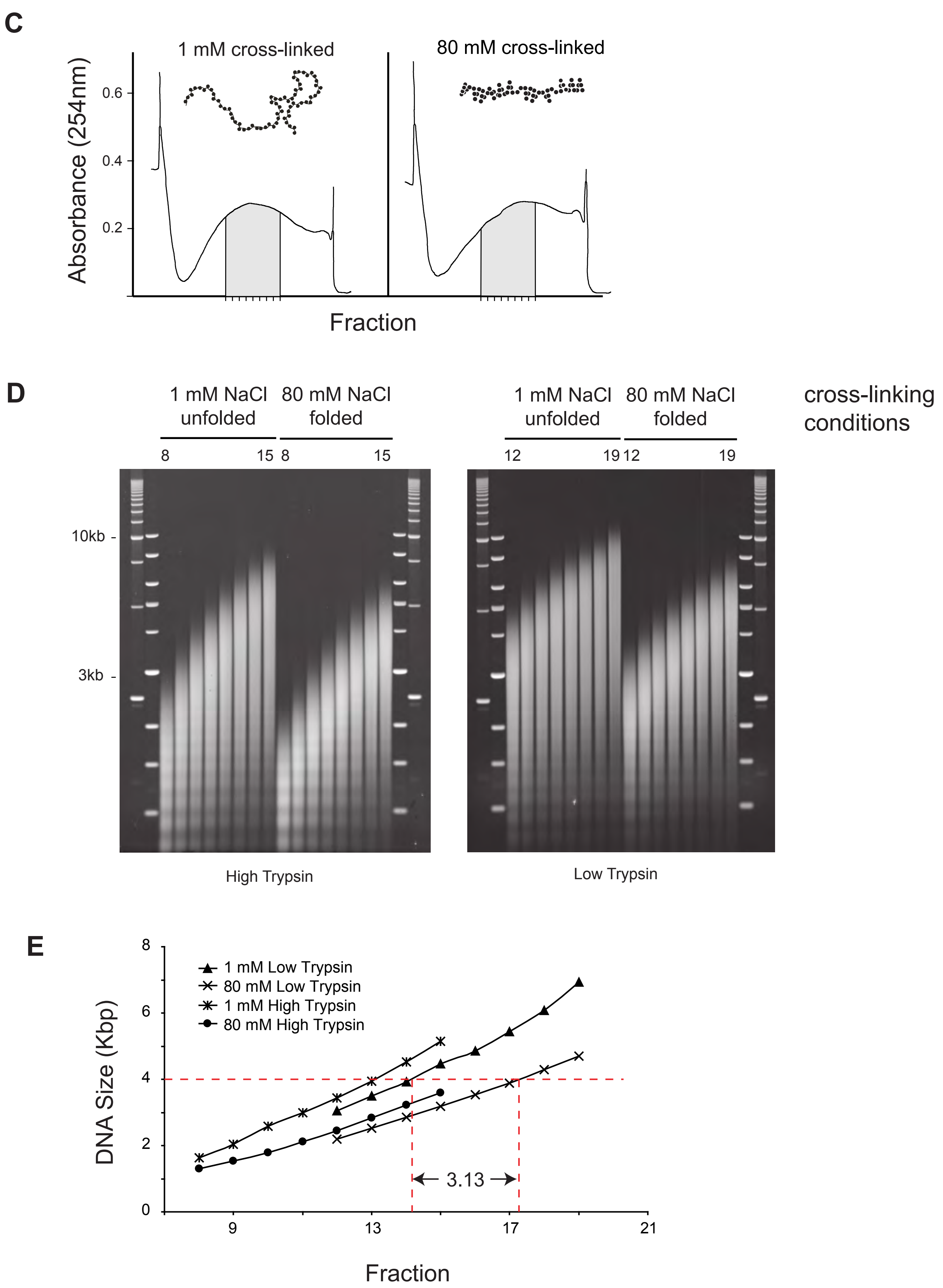
C
1 mM cross-linked
80 mM cross-linked
Absorbance (254nm)
0.6
0.4
0.2
Fraction
D
1 mM NaCl unfolded
80 mM NaCl folded
1 mM NaCl unfolded
80 mM NaCl folded
cross-linking conditions
8
15
8
15
12
19
12
19
10kb
3kb
High Trypsin
Low Trypsin
E
1 mM Low Trypsin
80 mM Low Trypsin
1 mM High Trypsin
80 mM High Trypsin
DNA Size (Kbp)
8
6
4
2
0
9
13
17
21
3.13
Fraction

Figure S3

Figure S3 (related to Figure 6). Differently folded, cross-linked, and trypsin digested NIH3T3 chromatin fibres have different sedimentation rates.

A. Schematic showing the isolation of soluble chromatin fibres from cells. Chromatin fibres were either folded in 80 mM NaCl or unfolded in 1 mM NaCl. Chromatin structure was fixed by extensive formaldehyde cross-linking introducing intramolecular bonds. After cross-linking cross-linker was remove by dialysis, trypsin digested to remove tail-to-tail interactions and solubilised in SDS. Samples were then fractionated on a sucrose gradient in low salt (1 mM NaCl).

B. Coomassie stained protein gel showing nuclear proteins in total NIH3T3 nuclei (lane 1), chromatin proteins before cross-linking (lanes 2 and 3), after cross-linking (lanes 4 and 5) and after trypsin digestion (lanes 6 and 7).

C. Cross-linked chromatin isolated from NIH3T3 cells, and folded in either low (1 mM; unfolded) or high salt (80 mM NaCl; folded) and fractionated in an isokinetic sucrose gradient in the same centrifuge in buffer containing 1 mM NaCl. Graphs showing the absorbance (254 nm) across the sucrose gradients (top of gradient is left side).

D. DNA purified from individual fractions from each gradient (grey area in C) was fractionated on an agarose gel, stained with ethidium bromide and scanned on a laser scanner. Left, samples digested with high levels of trypsin, Right, samples digested with low levels of trypsin.

E. Densitometry of ethidium bromide staining for individual lanes shown in panel D to identify the size of peak fractions. Graph shows relationship between DNA size and fraction (sedimentation rate).

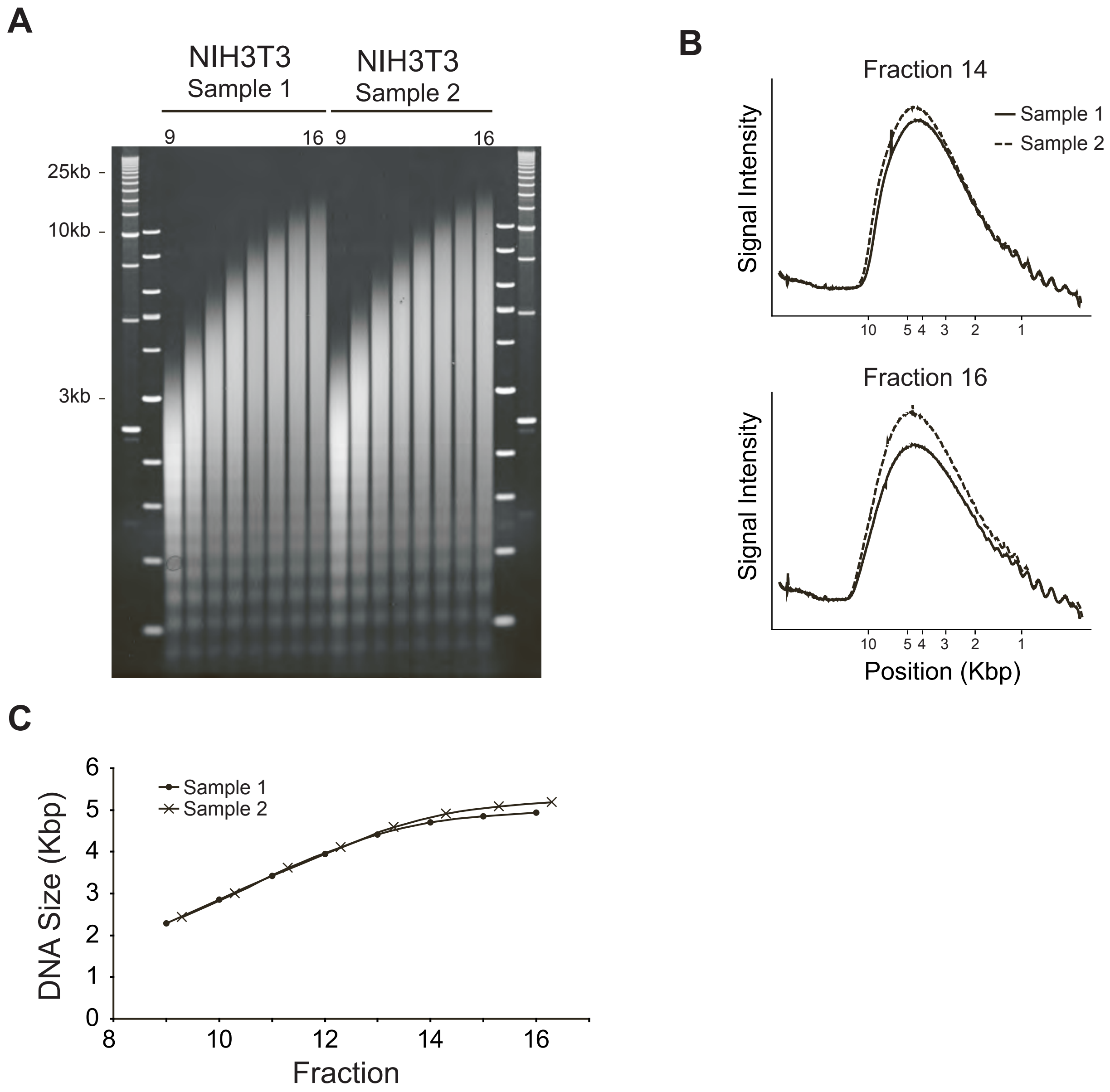

Figure S4 (related to Figure 6)

Reproducibility of extracting cross-linked, trypsin digested NIH3T3 chromatin samples

A. Cross-linked and trypsin digested chromatin was isolated from independent samples of NIH3T3 cells and fractionated on sucrose gradients in low (1 mM) NaCl. After fractionation DNA was purified from individual samples and fractionated on an agarose gel, stained with EtBr and quantified on a laser scanner (FLA-5100).

B. Densitometry of DNA intestity in individual lanes from (top) fraction 14 and bottom (fraction 16.

C. Graph showing the relationship between DNA size and fraction number for the sedimentation of independent NIH3T3 chromatin samples.

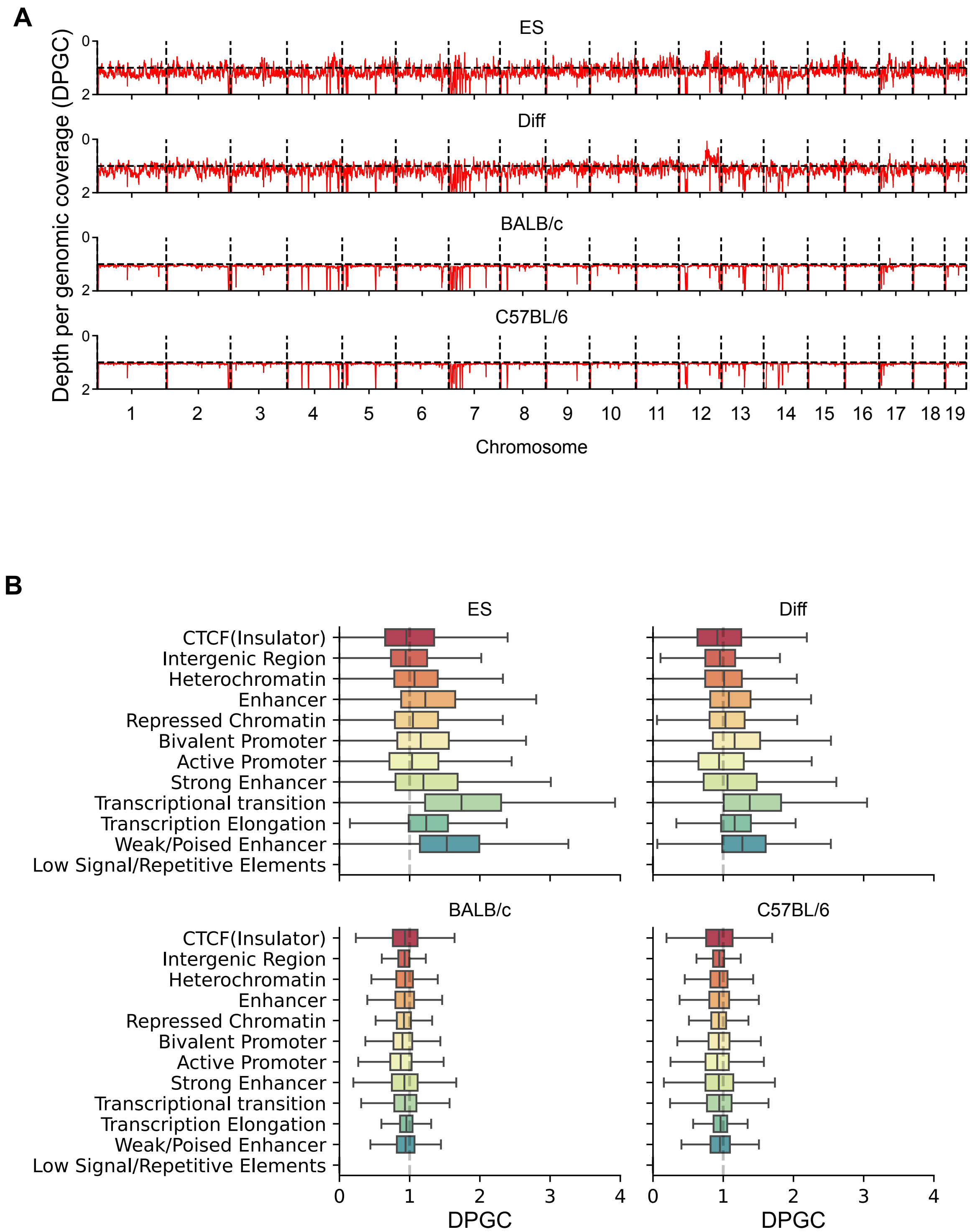

Figure S5 (related to Figure 6)

Representative release of soluble chromatin from cells

A. Soluble chromatin was purified from ES cells and differentiated ES cells and sequencec. Graph showing the distribution of released chromatin (in depth per genomic coverage) across each chromosome in the mouse genome, compared to the distribution of genomic DNA isolated from B cells from different mouse strains. Data shown is an average of two biological replicates, smoothed across 1 Mb windows.

B. Characterisation of chromatin into different genomic states using the chromHMM classification. Data shown is an average of two biological replicates.

Data S1

Original and uncropped gels for Fig1B, Fig 2A, Fig 3A, Fig 3B, Fig 4A, Fig 4C, Fig 4E, Fig 6B, Fig 6C, Fig 7B, Fig S1C, Fig S2B, Fig S3B, Fig S3D, Fig S4A.

Fig 1B

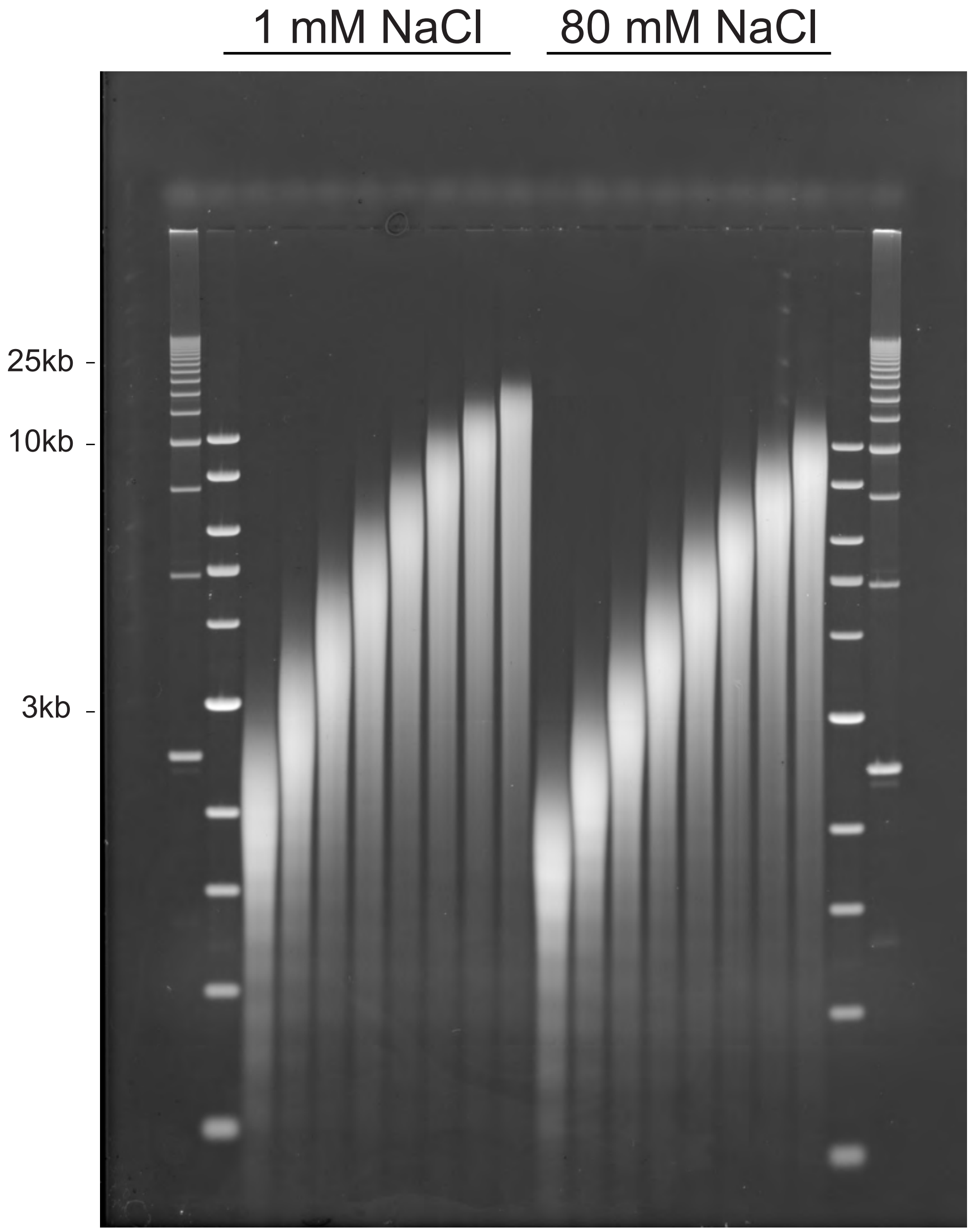

Fig 2A

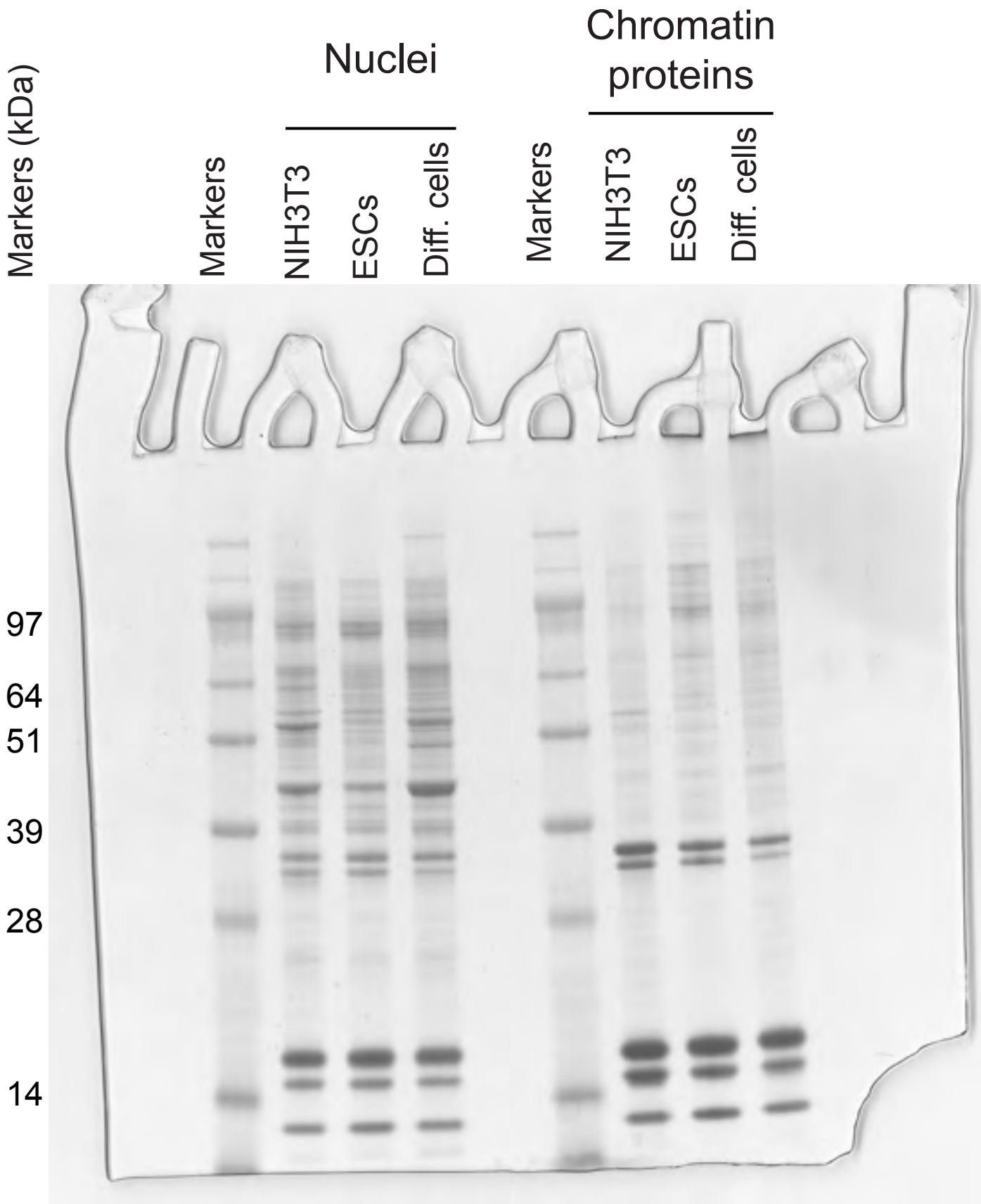
Markers (kDa)
Nuclei
Chromatin proteins
Markers
NIH3T3
ESCs
Diff. cells
Markers
NIH3T3
ESCs
Diff. cells
97
64
51
39
28
14

Fig 3A

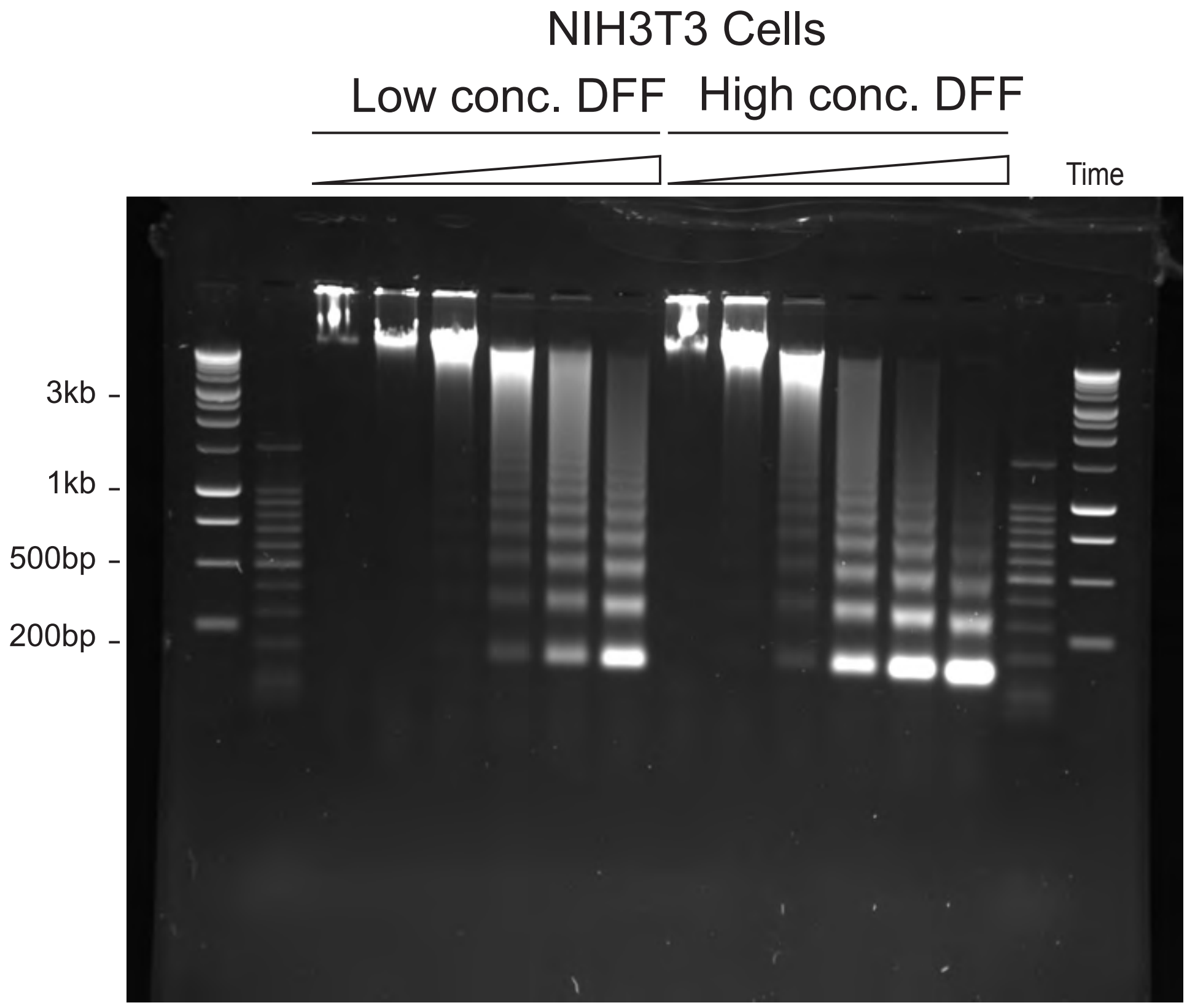

NIH3T3 Cells
Low conc. DFF
High conc. DFF
Time
3kb
1kb
500bp
200bp

Fig 3B

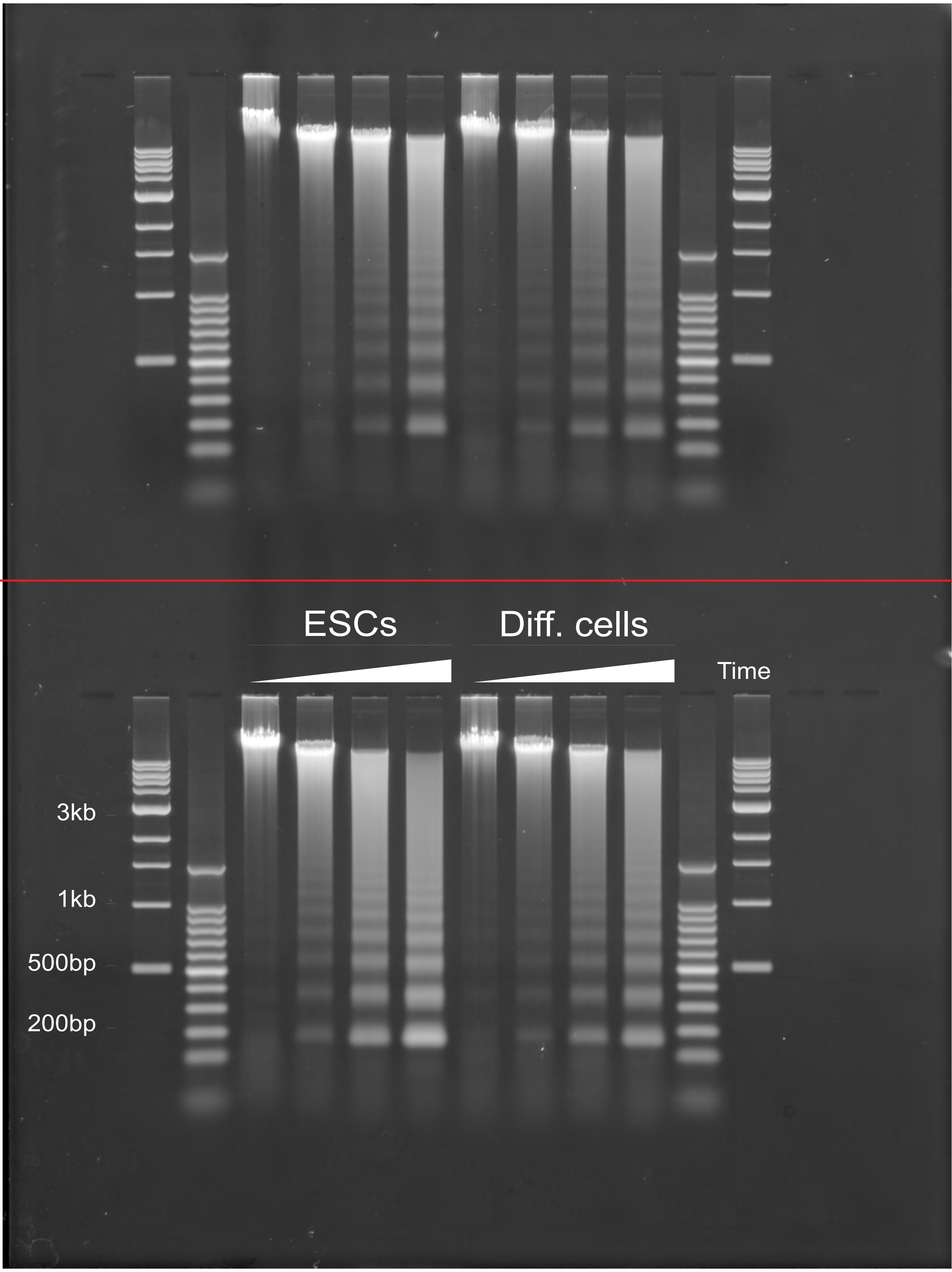

Included in manuscript

Fig 4A

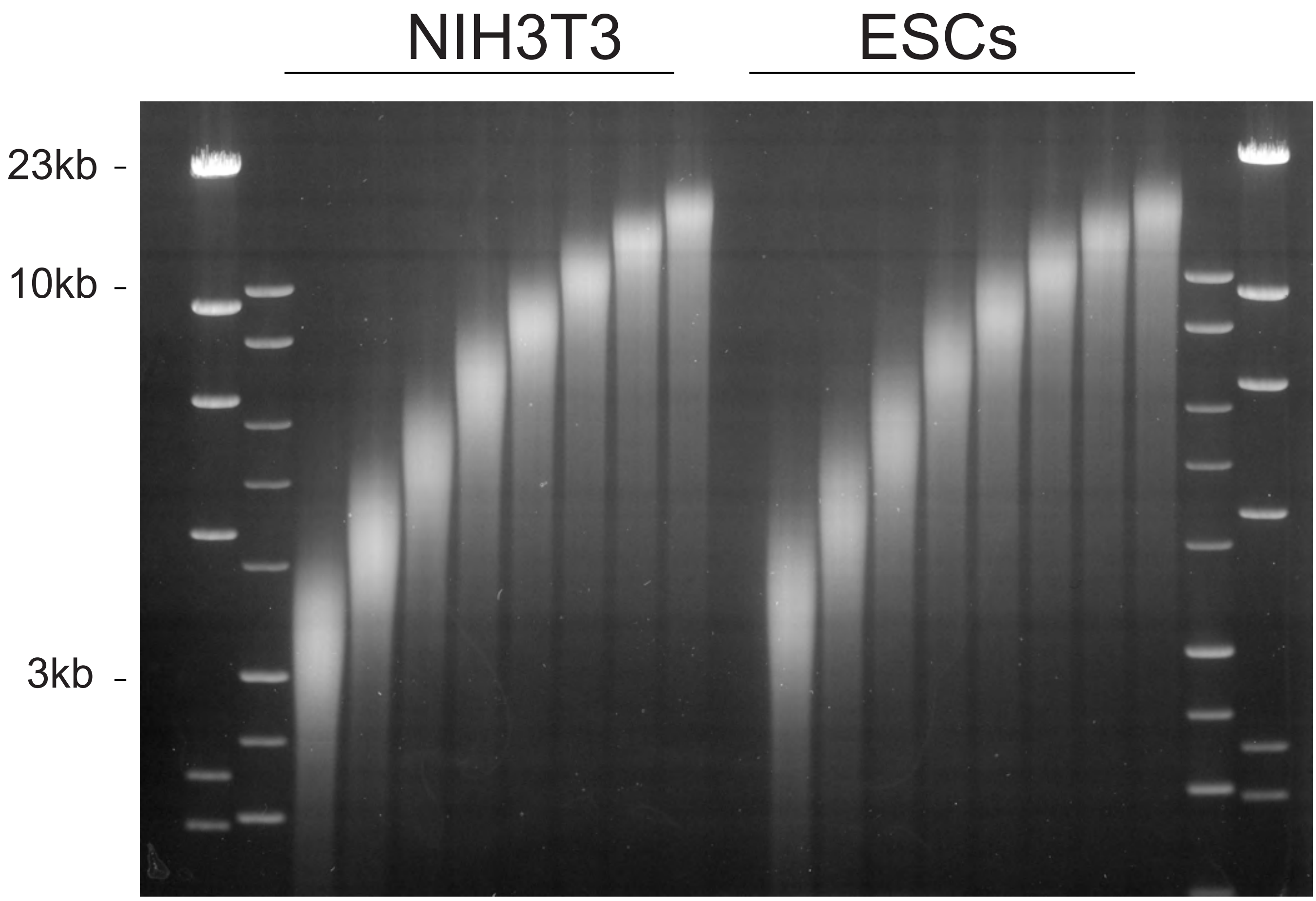

Fig 4C

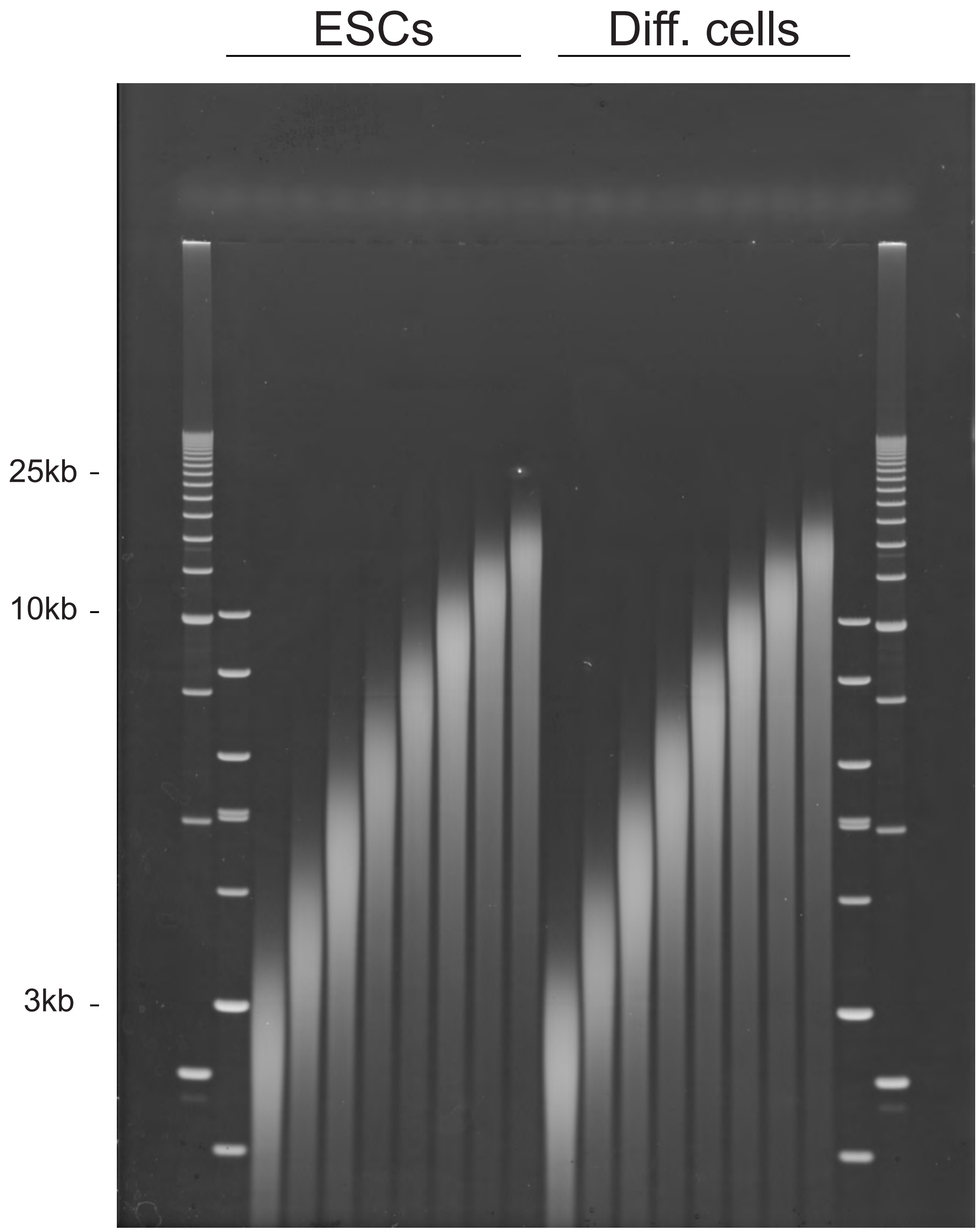

Fig 4E

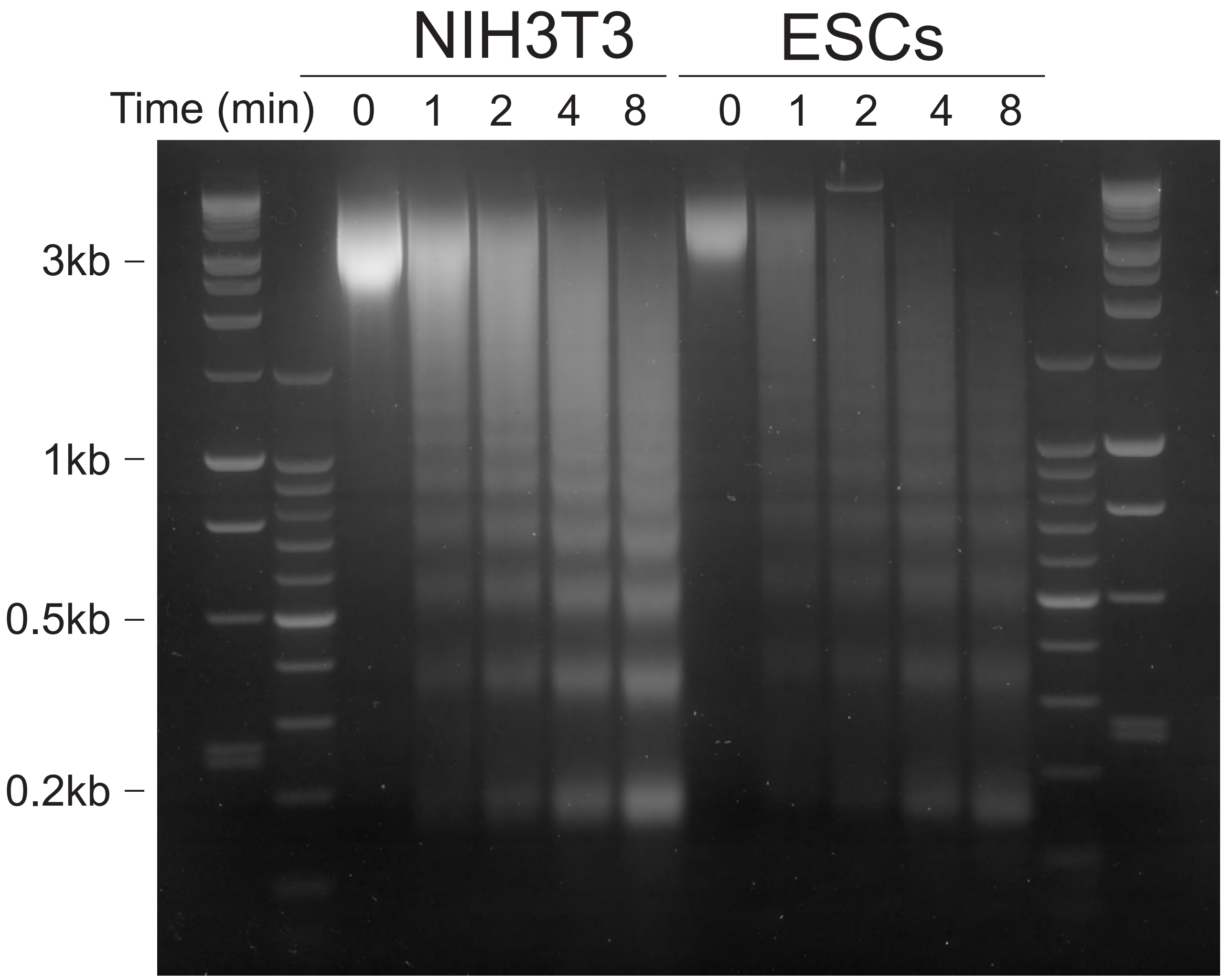

Fig 6B

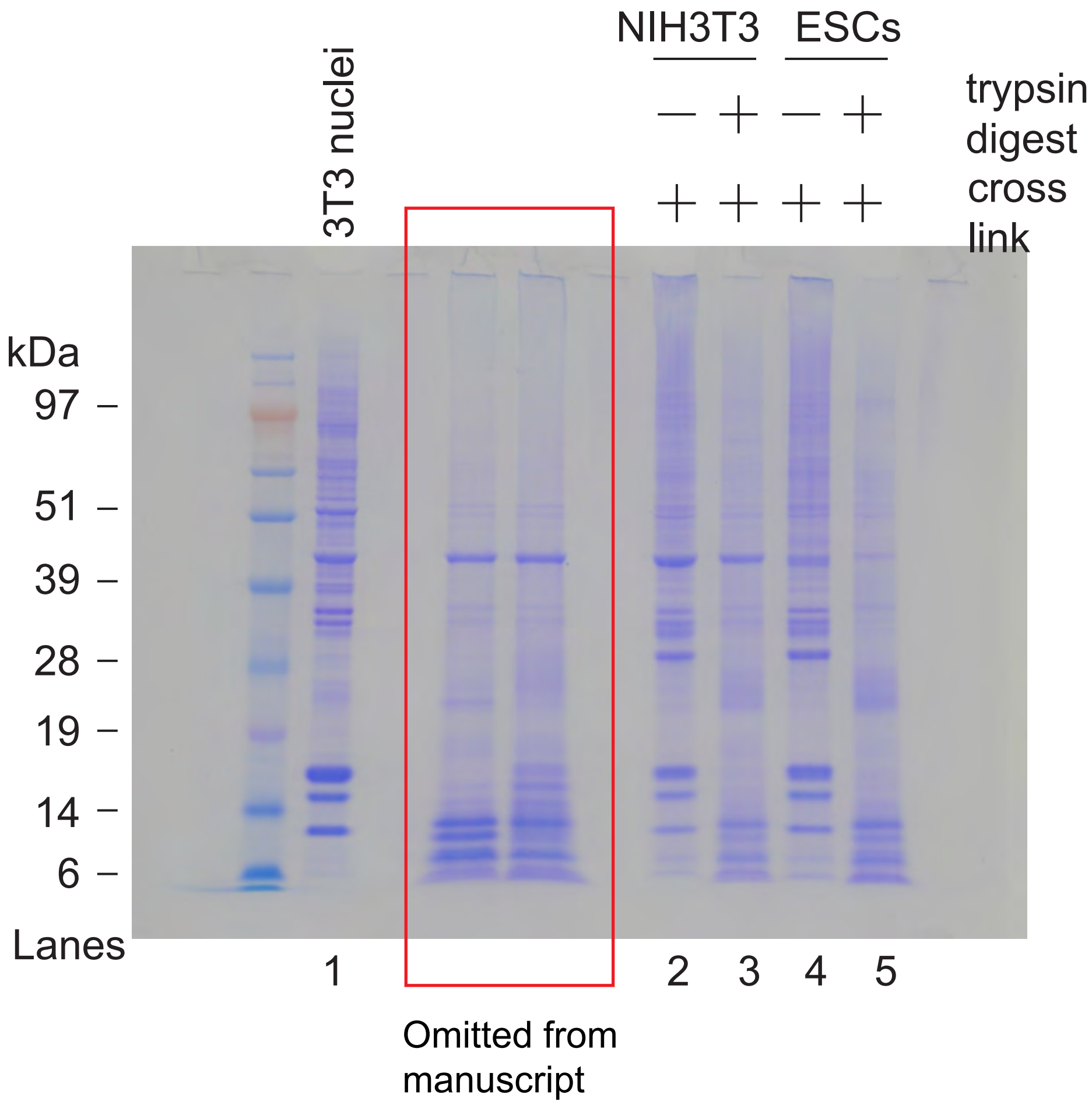
NIH3T3
ESCs
3T3 nuclei
trypsin digest
cross link
— + — +
+ + + +
kDa
97
51
39
28
19
14
6
Lanes
1
2
3
4
5
Omitted from manuscript

Fig 6C

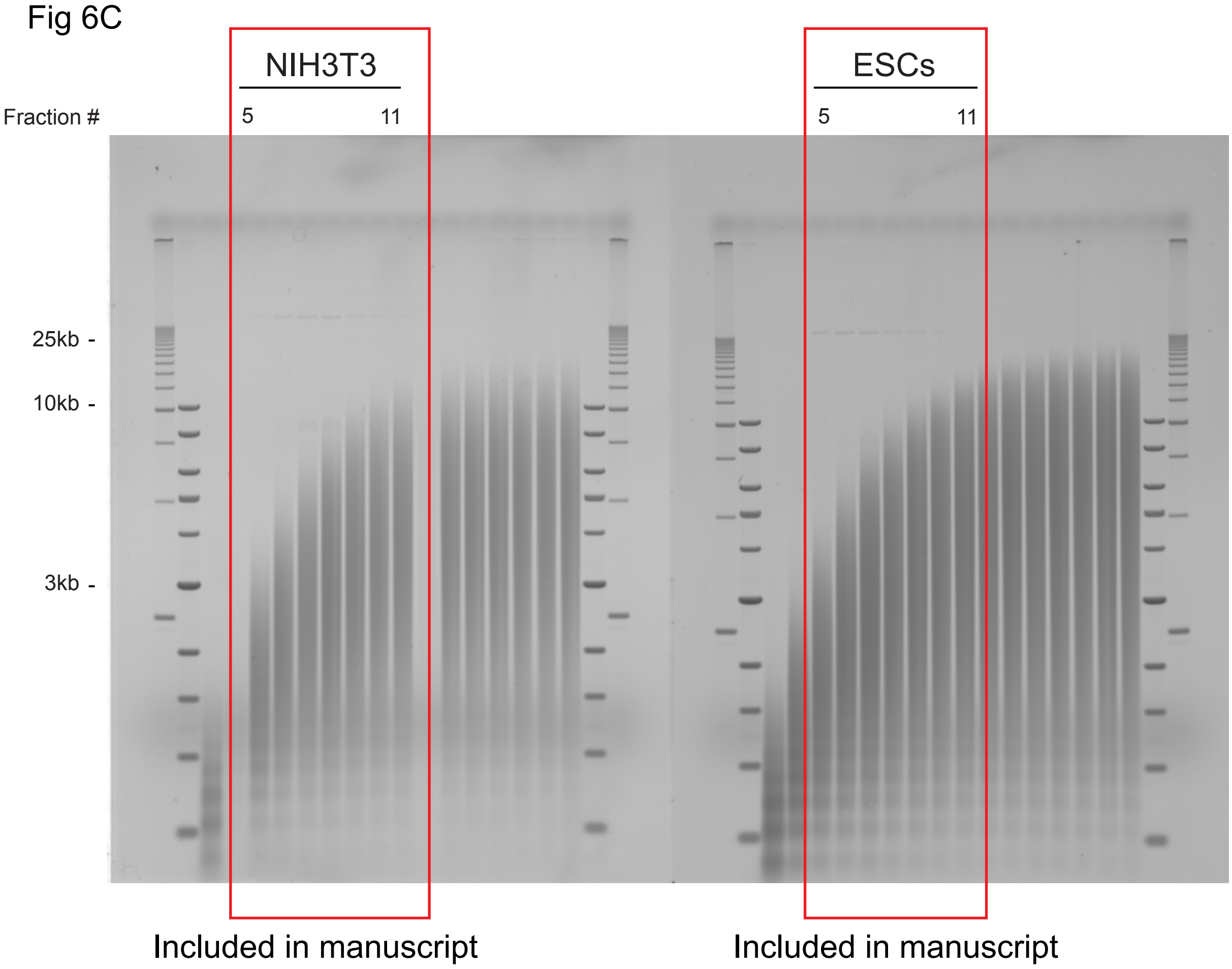
NIH3T3
ESCs
Fraction #
5
11
5
11
25kb -
10kb -
3kb -
Included in manuscript
Included in manuscript

Fig 7B

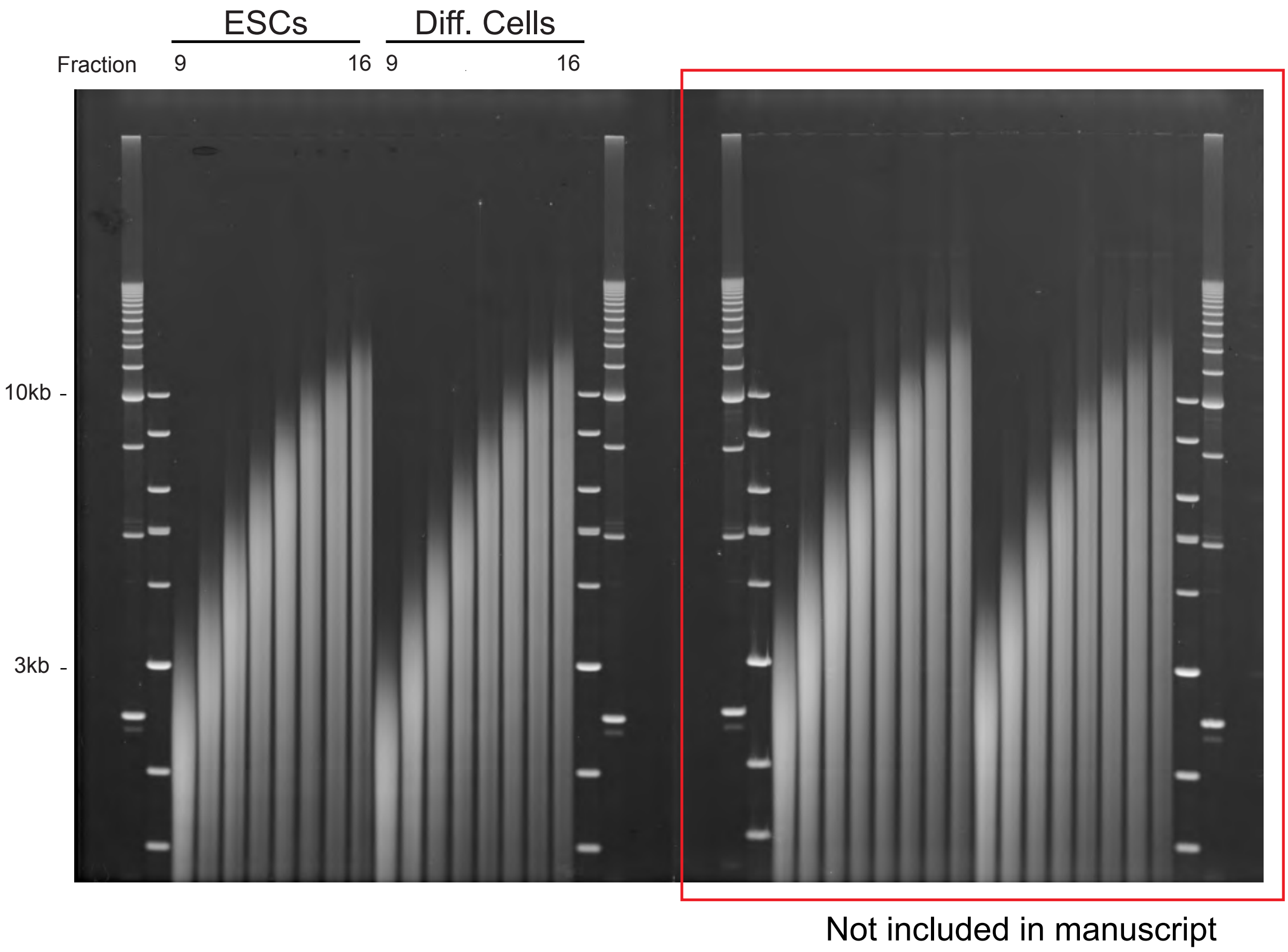
ESCs
Diff. Cells
Fraction
9
16
9
16
10kb -
3kb -
Not included in manuscript

Supp Fig 1C

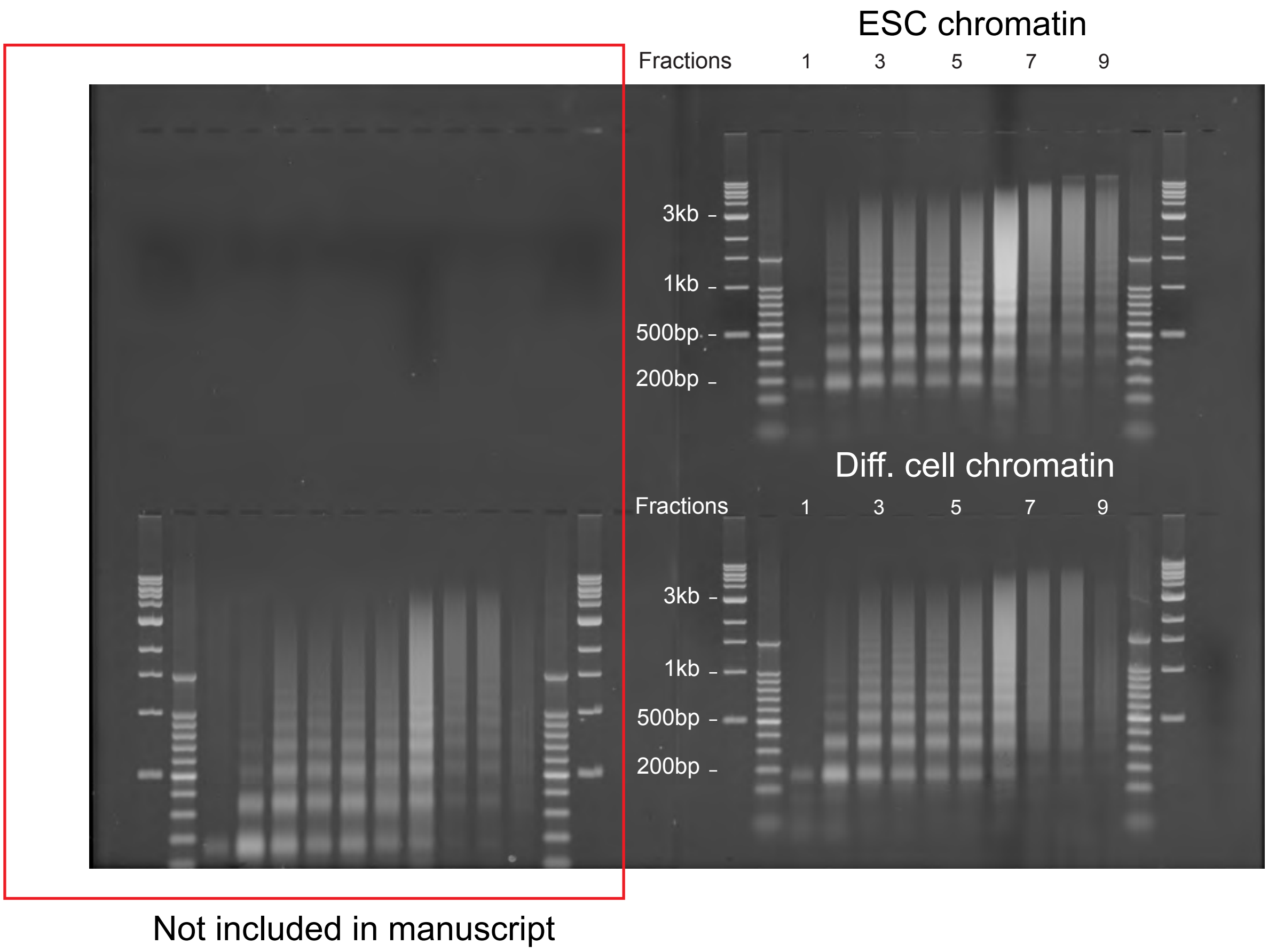
ESC chromatin
Fractions 1 3 5 7 9
3kb
1kb
500bp
200bp
Diff. cell chromatin
Fractions 1 3 5 7 9
3kb
1kb
500bp
200bp
Not included in manuscript

Supp Fig 2B

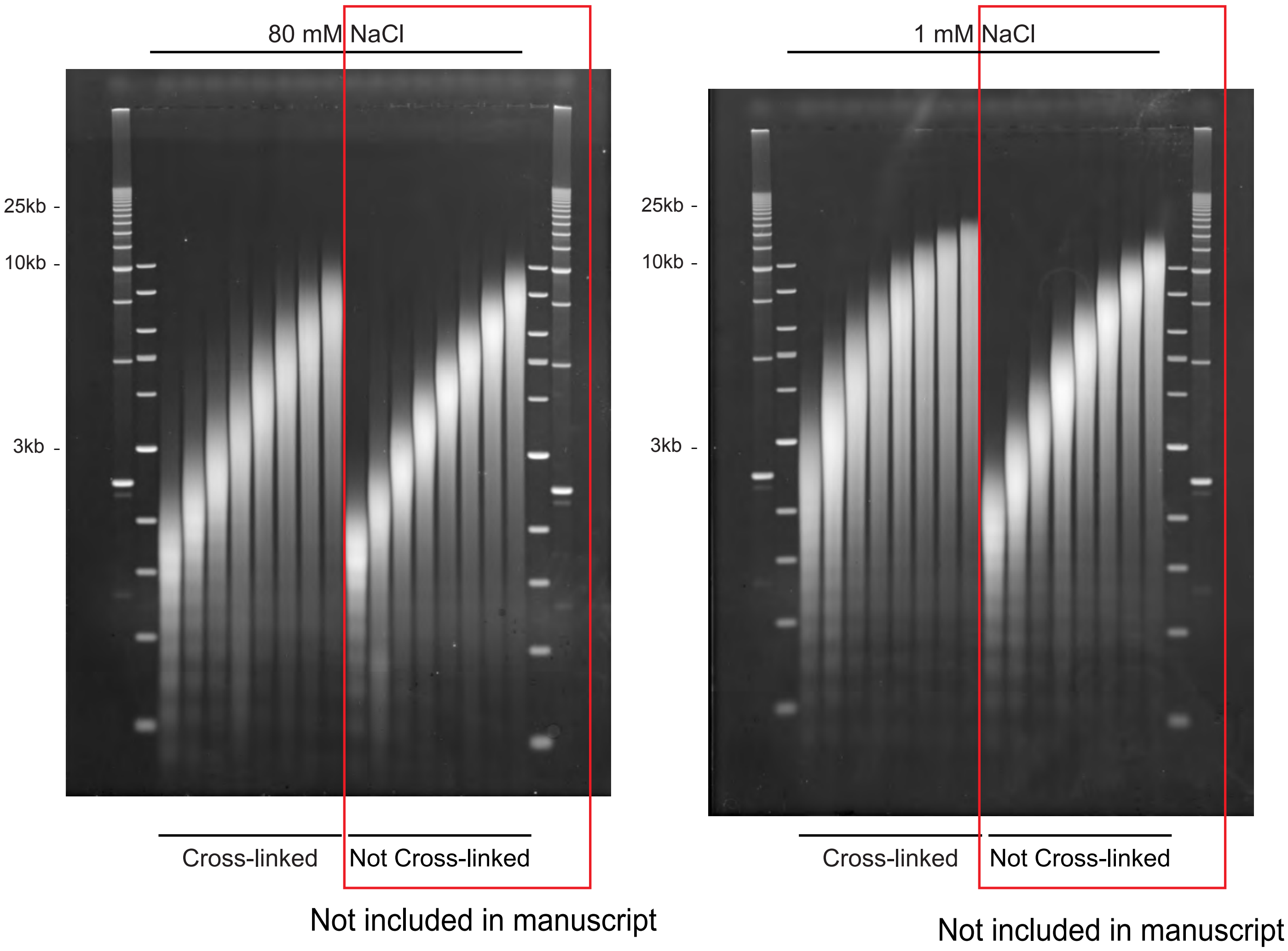
80 mM NaCl
25kb -
10kb -
3kb -
Cross-linked
Not Cross-linked
Not included in manuscript
1 mM NaCl
25kb -
10kb -
3kb -
Cross-linked
Not Cross-linked
Not included in manuscript

Supp Fig 3B

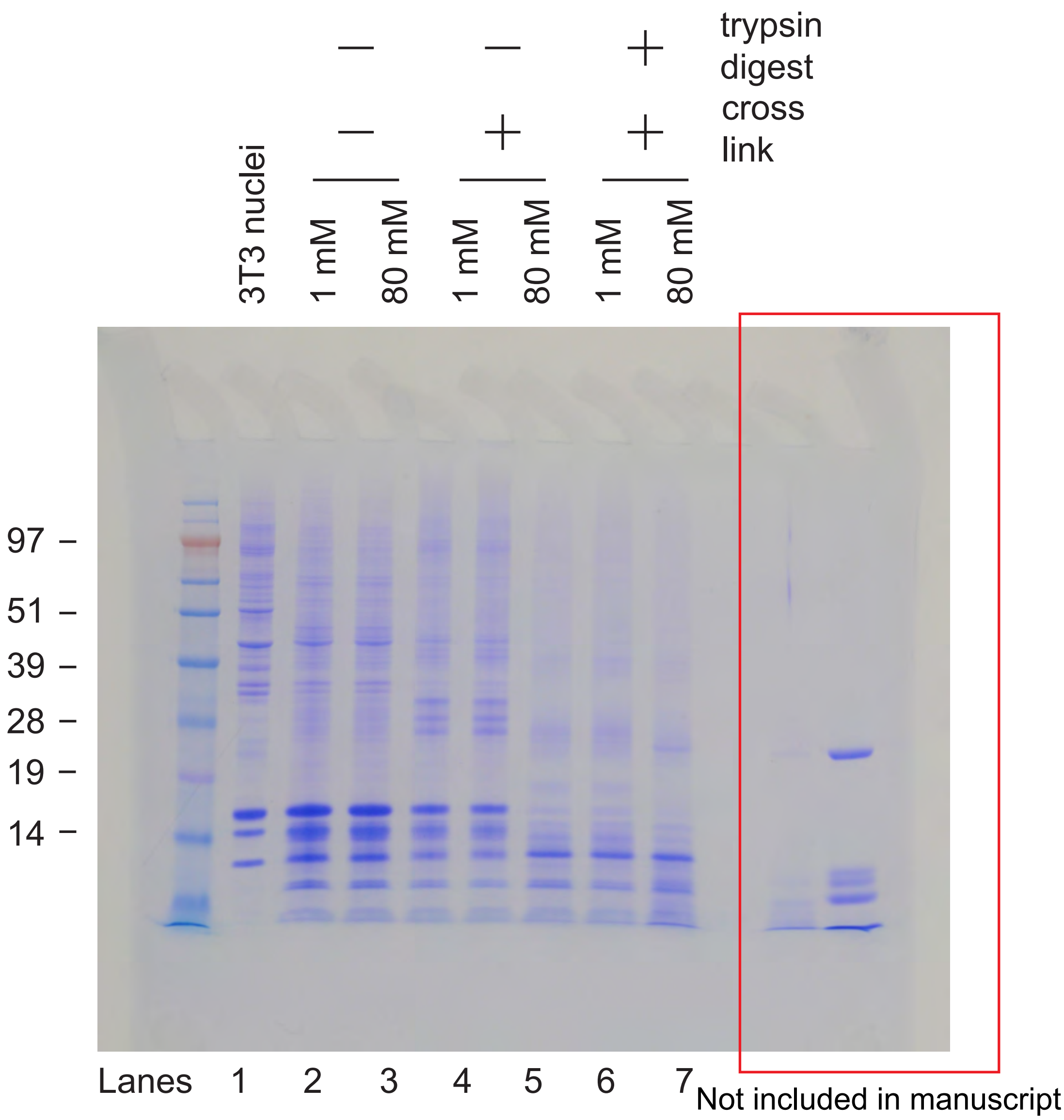

trypsin digest
cross link
3T3 nuclei
1 mM
80 mM
1 mM
80 mM
1 mM
80 mM
97
51
39
28
19
14
Lanes 1 2 3 4 5 6 7
Not included in manuscript

Supp Fig 3D

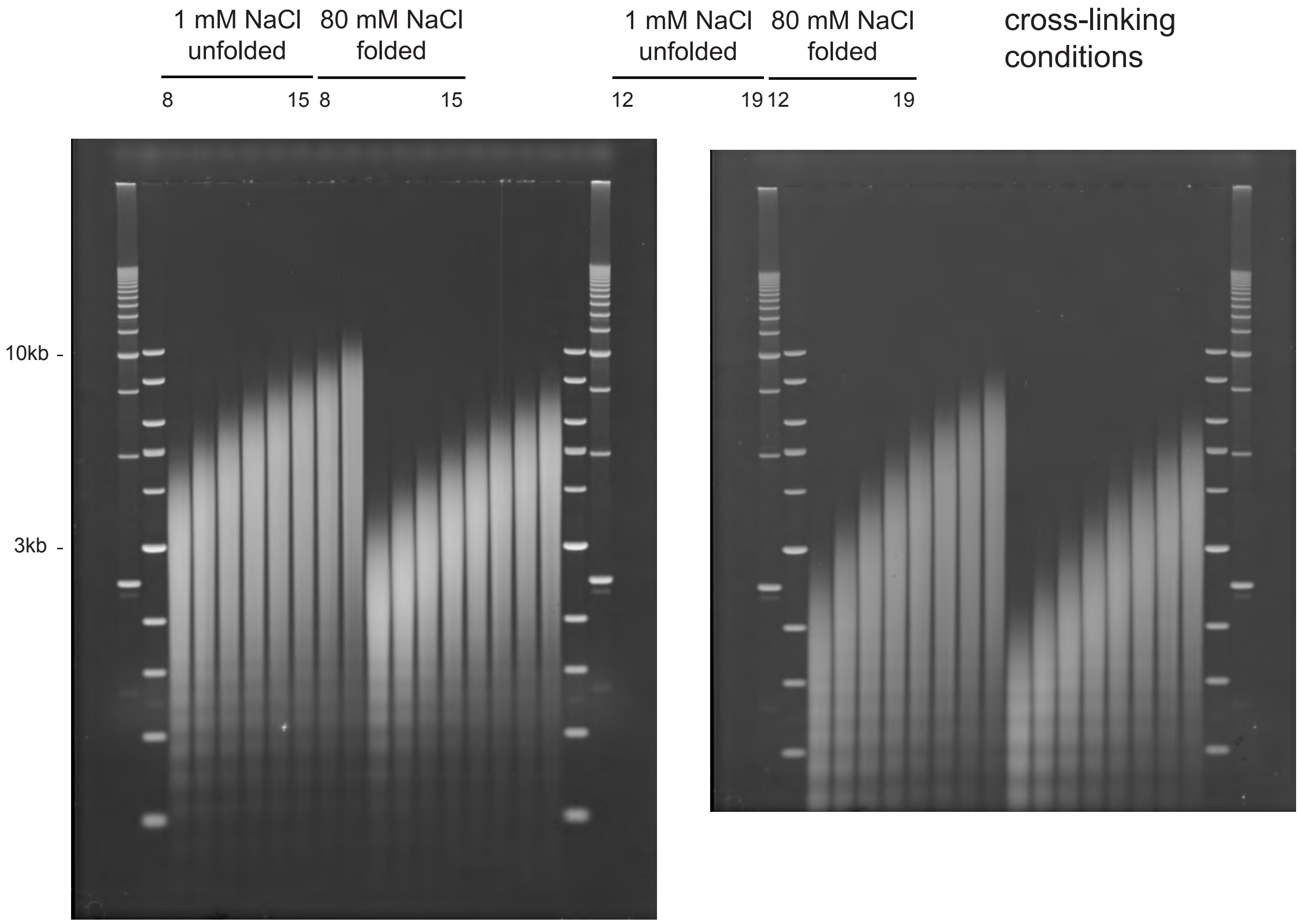

High Trypsin

Low Trypsin

Supp Fig 4A

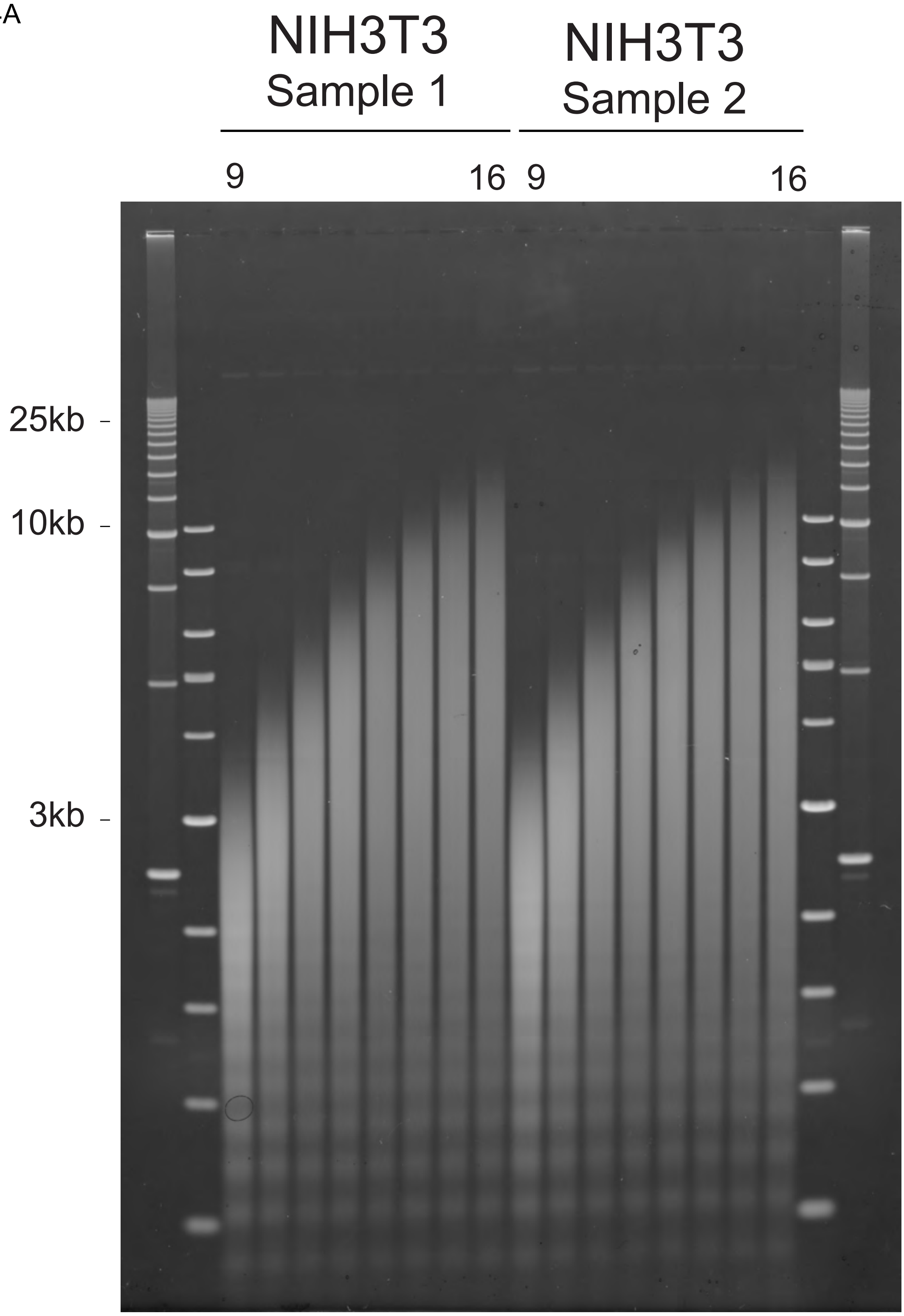

Supplement: Supplementary file 1 [file mmc1.pdf]
